# Dual-targeted hybrid nanovesicles coordinated bone-muscle regeneration via regulating the DUSP4/p38 MAPK pathway to reverse osteosarcopenia

**DOI:** 10.1186/s12951-026-04279-4

**Published:** 2026-03-16

**Authors:** Benchi Che, Yongzhi Cui, Zhengsheng Chen, Yanchun Gao, Lei Luo, Kaiwen zheng, Jiashuo Liu, Yu Xiang, Jiaqi Cheng, Yuanyuan Guo, Qing Li, Dehao Fu

**Affiliations:** 1 Department of Orthopedics, Institute of Microsurgery on Extremities, Shanghai Sixth People’s Hospital, Shanghai Jiao Tong University School of Medicine, Shanghai, 200233 China; 2 Department of Surgery, Hubei Provincial Hospital of Traditional Chinese Medicine, Wuhan, 430061 Hubei China; 3 Department of Pharmacy, Liyuan Hospital, Tongji Medical College, Huazhong University of Science and Technology, Wuhan, 430077 Hubei China

**Keywords:** Osteosarcopenia, Dual-targeted delivery, Extracellular vesicles, Hybrid vesicles, microRNA, p38 MAPK, DUSP4

## Abstract

Osteosarcopenia (OSP), a degenerative syndrome characterized by concurrent osteoporosis and sarcopenia, exhibits persistently high global prevalence due to aging and disuse, yet lacks targeted therapies. p38 MAPK plays a key regulatory role in musculoskeletal degeneration. Increased expression of DUSP4, a key inhibitor of the Mitogen-Activated Protein Kinase (MAPK) pathway, inhibits the phosphorylation of p38 MAPK. Thus, targeted delivery of microRNAs that inhibit DUSP4 expression can activate the p38 MAPK pathway and promote osteogenic and myogenic differentiation. However, the therapeutic efficacy of nucleic acid drugs critically depends on delivery systems with targeted specificity, stability, and biocompatibility. Therefore, we engineered a hybrid nanovesicle (miR@DT/iMNV) by fusing bone-muscle dual-targeting peptide-modified liposomes (miR@DT-Lipo) and iPSC-derived MSC-EVs (iMSC-EVs). The miR@DT/iMNV enables the dual-tissue targeted delivery of miR-206-5p (which is downregulated in OSP and can target and inhibit DUSP4) and exhibits excellent biocompatibility. Results demonstrated that miR@DT/iMNV enhances osteogenic/myogenic capacities of stem cells, modulates macrophage phenotypes, restores mitochondrial function, and increases bone/muscle mass in a disused OSP murine model. This dual-targeted, multi-mechanistic strategy presents an innovative therapeutic approach for OSP.

**Graphical abstract:**

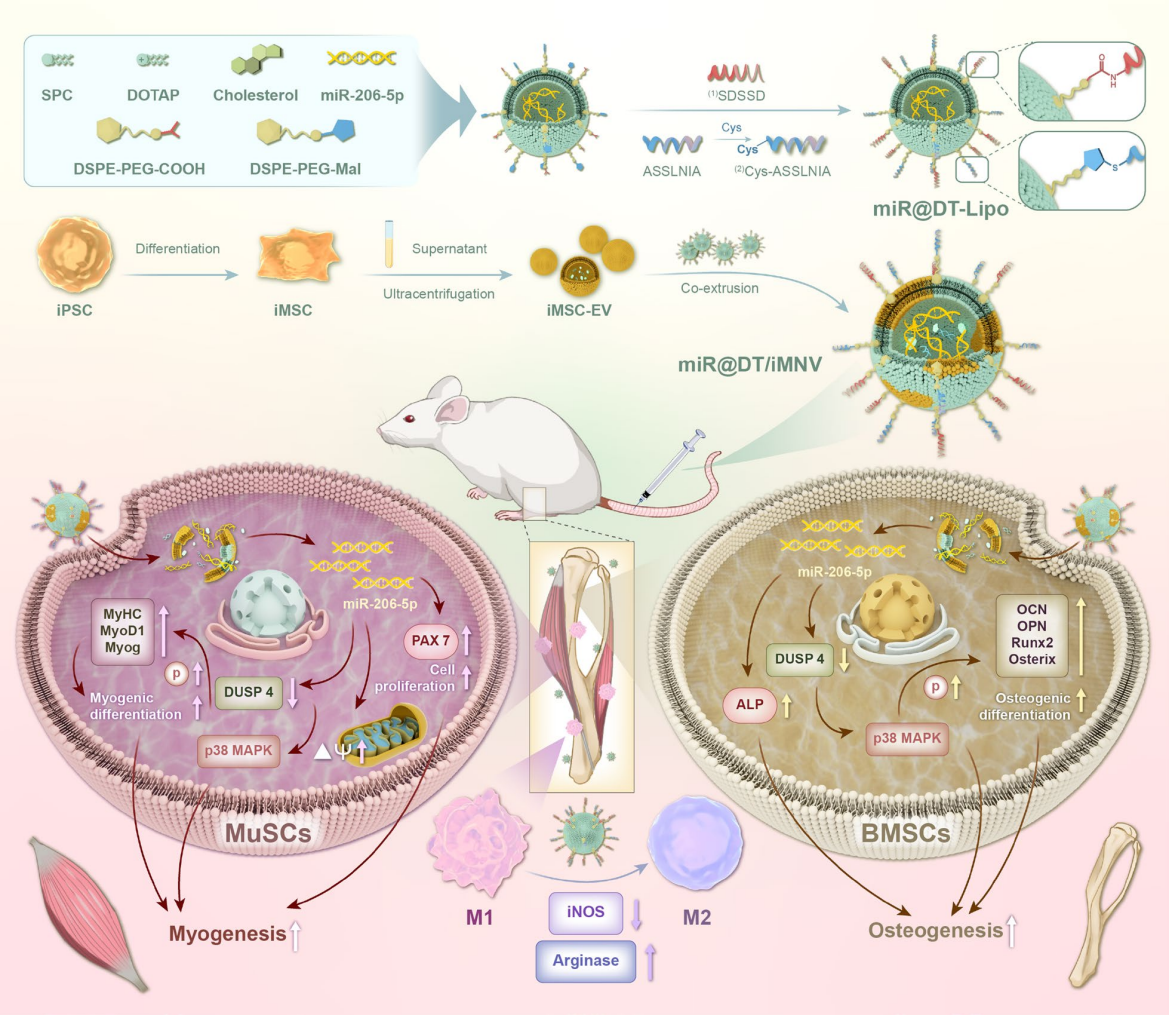

**Supplementary Information:**

The online version contains supplementary material available at 10.1186/s12951-026-04279-4.

## Introduction

Osteosarcopenia (OSP) is defined as the co-occurrence of osteopenia/osteoporosis (OP) and sarcopenia (SP), characterized by a decline in both bone and muscle mass [1]. Compared to isolated muscle or bone disorders, OSP patients are more prone to adverse outcomes such as falls and fractures, which severely impact their quality of life and increase mortality risk [2, 3]. Epidemiological studies indicate that the prevalence of OSP remains high across different regions globally, exceeding 20% in Asia, Oceania, and other areas, while the lowest incidence, observed in Europe, still reaches 10.9% [4, 5]. Beyond aging, long-term disuse—including exposure to low-gravity environments (e.g., spaceflight, as reported by NASA to induce OP and SP) [6], insufficient physical activity (per the 2024 WHO report, ~ 1.8 billion people globally fail to meet recommended activity levels), and prolonged bed rest/immobilization in post-surgical patients—constitutes a prominent contributing factor to OSP [7]. Notably, post-surgical immobilization specifically leads to rapid depletion of bone and muscle mass, further highlighting the clinical relevance of disuse-induced OSP [8, 9]. In summary, this disuse-induced OSP has posed an increasingly severe hazard to human beings. Currently, treatment for disuse-induced OSP is limited to exercise training or nutritional supplementation, with no targeted pharmacological options available.

During the pathogenesis of disuse-induced OSP, the p38 Mitogen-Activated Protein Kinase (MAPK) pathway is critical [10]. It is involved in key physiological and pathological processes such as cell survival, apoptosis, inflammatory response, differentiation, and metabolic balance, and exerts a core regulatory role in the physiological development, homeostasis maintenance, and injury repair of muscles and bones [11–14]. Studies have shown that activating the p38 MAPK pathway can significantly promote osteogenic and myogenic differentiation [15–18]. Inhibited phosphorylation of the p38 MAPK pathway disrupts the normal metabolism and function of muscle and bone cells, leading to osteomuscular metabolic imbalance, chronic inflammation, and differentiation disorders. The inactivated p38 MAPK pathway can also induce mitochondrial dysfunction in cells, resulting in reduced oxidative phosphorylation efficiency and oxidative stress damage, which impairs the regenerative function of muscle and bone stem/progenitor cells [19, 20]. In summary, under pathological conditions, abnormal function of the p38 MAPK pathway has become a key driver of disuse-induced OSP. How to activate and maintain its normal function is expected to be a critical strategy for improving degenerative lesions of musculoskeletal tissues.

Dual Specificity Phosphatase 4 (DUSP4) is a core member of the dual-specificity phosphatase family, whose primary function is to precisely regulate intracellular signaling pathways via dephosphorylation [21]. As a key inhibitor of the MAPK pathway, DUSP4 has been the focus of numerous breakthrough studies in recent years [22]. Research confirms that DUSP4 directly blocks the activation of the p38 MAPK pathway by inhibiting the phosphorylation of p38 MAPK [23, 24]. Thus, how to abrogate the inhibitory effect of DUSP4 on p38 MAPK has become a pivotal scientific question in the field of OSP treatment. Against this backdrop, our research focus shifted to microRNAs (miRs)—short non-coding RNAs, typically 21–23 nucleotides in length—that precisely suppress post-transcriptional gene expression by complementary base pairing with target messenger RNAs (mRNAs) [25]. miR-206-5p was identified as a potential candidate, whose expression is significantly downregulated in the pathological context of OSP [26]. Recent studies further validate that exogenous intervention or indirect modulation of miR-206-5p expression can effectively promote the regeneration of bone, highlighting its potential as a therapeutic target [27]. Although the regulatory function of miR-206-5p in muscle remains unclear, its precursor, miR-206, has been identified as a key regulatory factor in skeletal muscle development and confirmed to be enriched in myogenic stem/progenitor cells [28, 29]. This finding suggests that targeted delivery of miR-206-5p may enable the regulation of the DUSP4/p38 MAPK pathway, thereby achieving the goal of treating OSP. However, according to the current studies, how to deliver miR-206-5p to its target site more effectively remains a major challenge.

In earlier studies, single-component vesicles (such as liposomes and extracellular vesicles (EVs)) have shown certain application potential in drug delivery [30–33]. However, they are limited by their own performance defects—for instance, liposomes have insufficient biocompatibility, while EVs are difficult to modify and their activity is easily disrupted. These issues make it hard for them to meet the practical needs in complex biological environments. To address this, researchers have begun to explore the “hybrid strategy”: rational design and combination of assembly units with distinct properties to construct hybrid vesicles that integrate multi-component advantages [34, 35]. For instance, liposomes—which offer high flexibility in design and synthesis—can be utilized for targeted modifications and drug encapsulation [36]. By hybridizing them with biologically derived EVs, the resulting hybrid vesicles can further capitalize on the diverse active molecules naturally present in EVs, as well as their superior biocompatibility, leading to enhanced therapeutic efficacy [37–39]. Moreover, the physical extrusion method circumvents the destruction and loss of active molecules that often occur in traditionally engineered EVs, making this approach truly multi-beneficial. Hybrid vesicles generated by EV-liposome fusion synergistically combine both systems’ strengths while mitigating weaknesses, providing a promising strategy for complex syndromes like OSP.

In this study, we pioneered a dual-targeting hybrid nanovesicle (miR@DT/iMNV) by fusing muscle/bone dual-targeting peptide-modified liposomes loaded with miR-206-5p (miR@DT-Lipo) and extracellular vesicles from iPSC-derived mesenchymal stem cells (iMSC-EVs). This hybrid vesicle, for the first time, achieves dual-targeting of bone and muscle tissues, while efficiently encapsulating miR-206-5p, and retaining the bioactive components and biocompatibility of iMSC-EVs. Cellular assays demonstrated enhanced osteogenic differentiation in BMSCs and myogenic differentiation in muscle satellite cells (MuSCs), alongside conferring multiple therapeutic benefits, including anti-inflammatory effects and so on. In an OSP mouse model, miR@DT/iMNV alleviated bone and muscle loss. Mechanistically, miR-206-5p targets DUSP4 to activate p38 MAPK phosphorylation—a “shared pathway” simultaneously enhancing myogenesis and osteogenesis—thus embodying a dual-pathology intervention strategy. In conclusion, miR@DT/iMNV exhibits excellent therapeutic effects, highlighting its translational potential for OSP treatment.

## Results

### The ability of miR-206-5p to target DUSP4 and its functional prediction

**Fig. 1:**
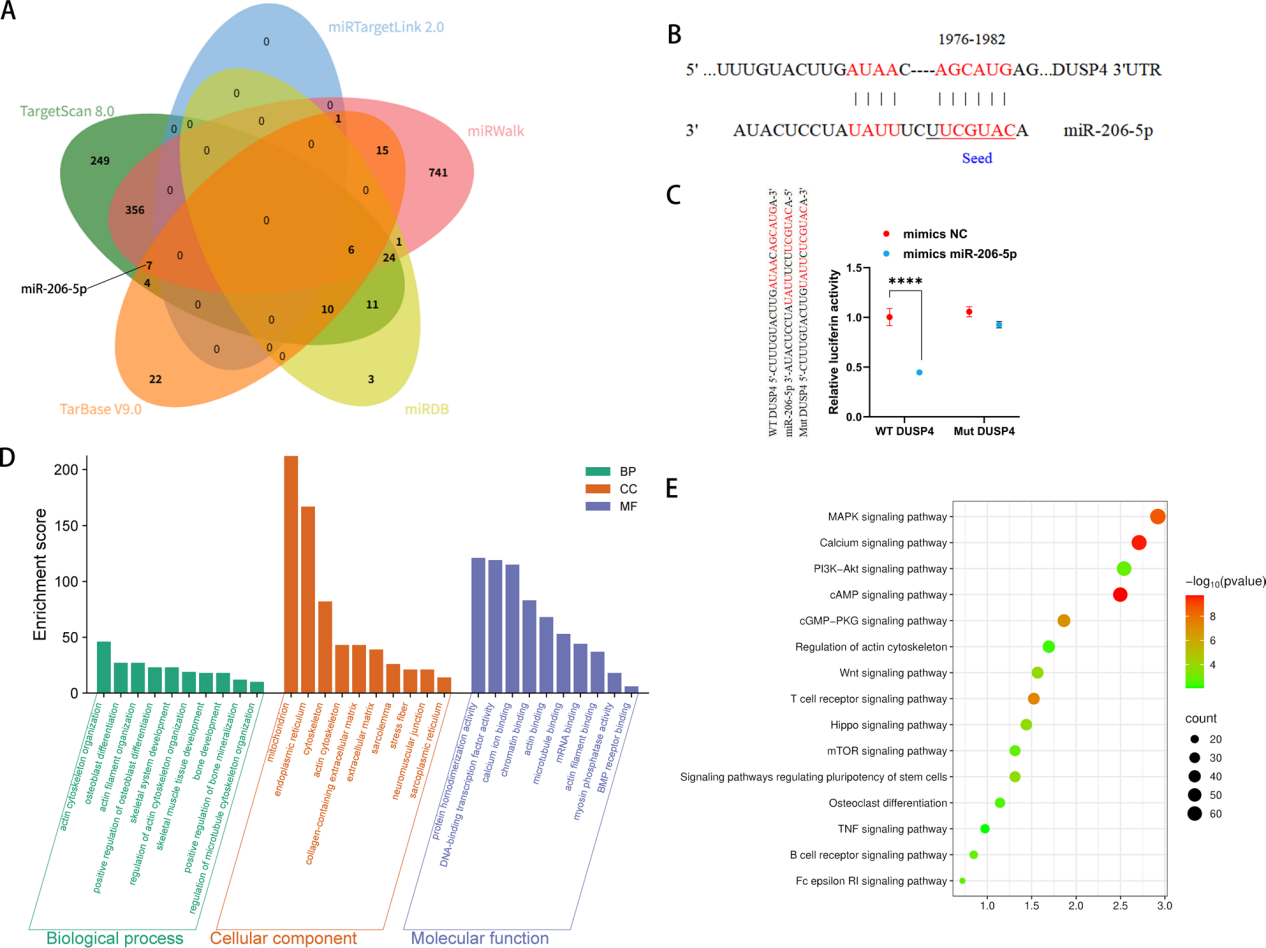
The ability of miR-206-5p to target DUSP4 and its functional prediction. **(A)** Venn diagram of miRs predicted to bind DUSP4 by multiple databases. **(B)** Schematic diagram of the binding site between DUSP4 and miR-206-5p. **(C)** The luciferase activity of the WT DUSP4 and Mut DUSP4 in 293 T cells treated with mimics NC or mimics miR-206-5p. **(D)** Enrichment plots of BP, CC, and MF derived from GO analysis of miR-206-5p. **(E)** Dot plot of potential signaling pathways of miR-206-5p via KEGG analysis

Activation of the p38 MAPK pathway can treat osteoporosis and sarcopenia through multiple mechanisms, providing important insights for the treatment of osteosarcopenia [13, 14, 16, 18]. As a key inhibitor of the MAPK pathway, DUSP4 has been shown to inactivate p38 MAPK by inhibiting its phosphorylation, thereby negatively regulating osteogenic and myogenic processes [24]. To target the inhibition of DUSP4 expression, we first used multiple databases(e.g., TargetScan, miRDB, miRTartgetLink, miRWalk and Tarbase) to predict miRs that target DUSP4 **(**Fig. 1A**)**. Among these results, we focused on miR-206-5p, which demonstrated binding affinity to DUSP4 in predictions across multiple databases. Additionally, miR-206-5p is downregulated in both osteoporosis and sarcopenia. Studies have also indicated that miR-206-5p plays a positive regulatory role in osteogenic and myogenic processes. To further confirm the regulatory relationship between miR-206-5p and DUSP4, we aligned their base sequences, which revealed that the seed sequence of miR-206-5p can complementarily pair with 3’-untranslated region (3’-UTR) of DUSP4 **(**Fig. 1B**)**. As shown in the dual-luciferase assays, miR-206-5p mimics significantly inhibited the luciferase activity of the WT DUSP4 **(**Fig. 1C**)**. Furthermore, we performed Gene Ontology (GO) and Kyoto Encyclopedia of Genes and Genomes (KEGG) analyses to characterize miR-206-5p, GO analysis revealed that miR-206-5p is enriched in biological processes (BP) including actin cytoskeleton organization, osteoblast differentiation, and skeletal muscle development; molecular functions (MF) such as calcium ion binding, chromatin binding, and BMP receptor binding; and cellular components (CC) targeting mitochondria, endoplasmic reticulum, and sarcoplasmic reticulum **(**Fig. 1D**)**. KEGG analysis further implicated miR-206-5p in the MAPK and calcium signaling pathways **(**Fig. 1E**)**. Collectively, these data establish miR-206-5p’s pivotal function in osteogenic and myogenic regulation.

### miR-206-5p improves the osteogenic and myogenic capacity by inhibiting DUSP4 to promote p38 MAPK phosphorylation

**Fig. 2:**
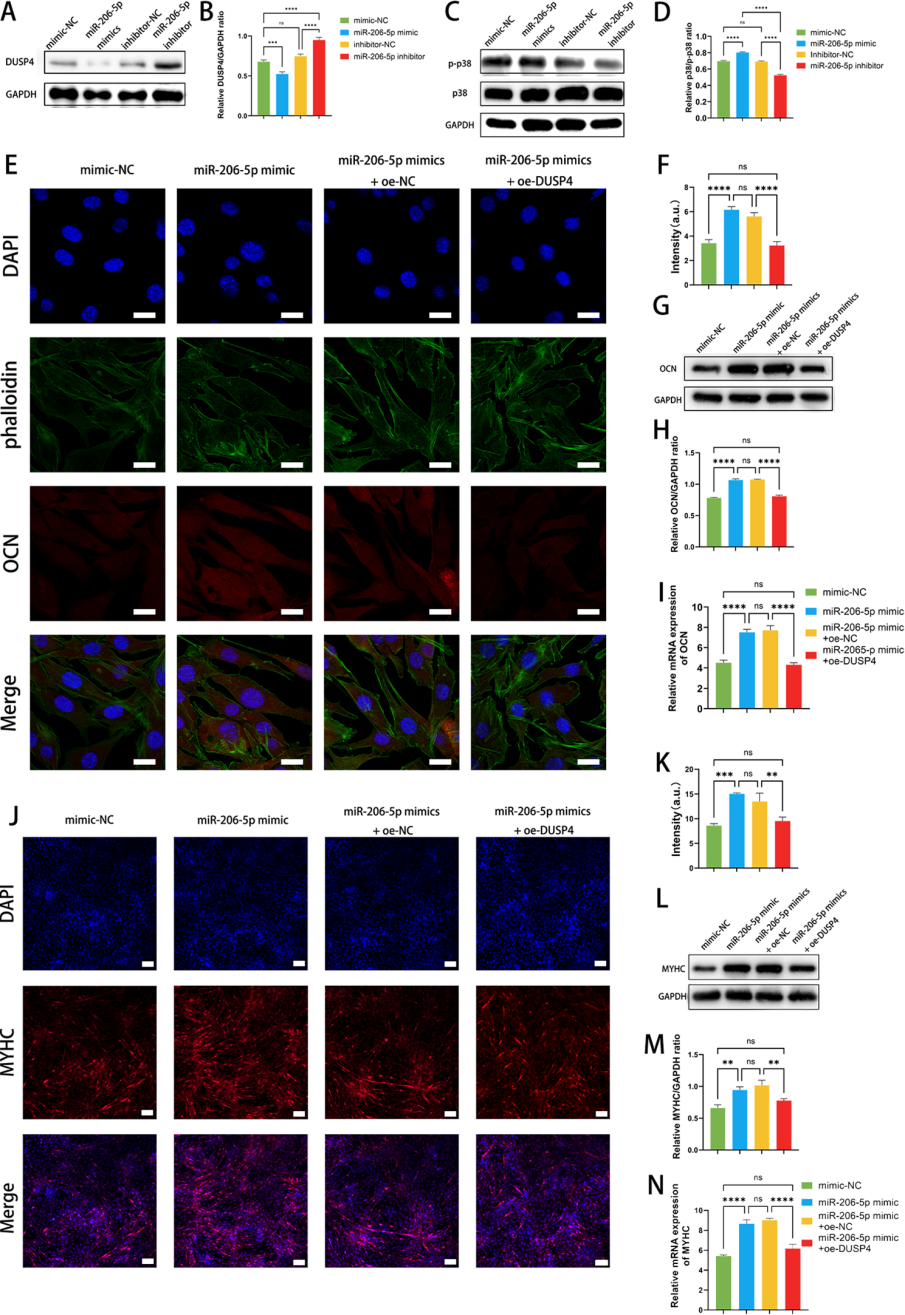
miR-206-5p improves the osteogenic and myogenic capacity by inhibiting DUSP4 to promote p38 MAPK phosphorylation. **(A)** The relative protein level of DUSP4 in BMSCs after different treatments. **(B)** Quantification of the relative gray level of the DUSP4 (*n* = 3). **(C)** The relative protein level of p38 and p-p38 in BMSCs after different treatments. **(D)** Quantification of the relative ratio of p-p38/p38 (*n* = 3). **(E)** Representative IF images showing expression of OCN by BMSCs after different treatments. Scale bar: 20 μm. **(F)** Quantitative analysis of the fluorescence intensity of OCN (*n* = 3). **(G)** The relative protein level of OCN in BMSCs after different treatments. **(H)** Quantification of the relative gray level of the OCN (*n* = 3). **(I)** Relative mRNA expression of OCN (*n* = 5). **(J)** Representative IF images showing expression of MYHC by MuSCs after different treatments. Scale bar: 100 μm. **(K)** Quantitative analysis of the fluorescence intensity of MYHC (*n* = 3). **(L)** The relative protein level of MYHC in MuSCs after different treatments. **(M)** Quantification of the relative gray level of the MYHC (*n* = 3). **(N)** Relative mRNA expression of MYHC (*n* = 5). *p < 0.05, **p < 0.01, ***p < 0.001, and ****p < 0.0001. Data are displayed as mean ± SD

To verify the direct regulatory relationship between miR-206-5p and DUSP4, we transfected miR-206-5p mimics, mimic negative control (NC), inhibitors, and inhibitor NC into BMSCs, respectively. Western blot analysis revealed that transfection with miR-206-5p mimics significantly suppressed DUSP4 expression (*p* < 0.001), whereas transfection with inhibitors reversed this inhibitory effect (*p* < 0.0001) **(**Fig. 2A-B**)**. Concurrently, miR-206-5p mimics markedly enhanced p38 MAPK phosphorylation (*p* < 0.0001), while inhibitors exerted the opposite effect (*p* < 0.0001) **(**Fig. 2C-D**)**. These results not only confirm the direct inhibitory effect of miR-206-5p on DUSP4 but also indicate that downregulated DUSP4 expression alleviates the inhibition of p38 MAPK phosphorylation.

To determine whether miR-206-5p-mediated promotion of osteogenic and myogenic differentiation depends on the DUSP4-p38 MAPK pathway, we constructed oe-NC and oe-DUSP4 overexpression plasmids. In BMSCs, immunofluorescence staining demonstrated that miR-206-5p mimics significantly upregulated the expression of the osteogenic marker protein osteocalcin (OCN) (*p* < 0.0001), whereas DUSP4 overexpression notably inhibited OCN expression (*p* < 0.0001) **(**Fig. 2E-F**)**. Western blot and RT-qPCR results further confirmed this regulatory axis: DUSP4 directly inhibits the osteogenic differentiation potential of BMSCs, while miR-206-5p alleviates this inhibitory effect by downregulating DUSP4 expression **(**Fig. 2G-I**)**. Similarly, in MuSC myogenic differentiation assays, miR-206-5p mimics significantly promoted myotube formation (*p* < 0.001), while DUSP4 overexpression inhibited this process (*p* < 0.01) **(**Fig. 2J-K**)**. Western blot and RT-qPCR analyses showed that DUSP4 overexpression resulted in a marked reduction in myosin heavy chain (MYHC) expression at both the protein and mRNA levels (*p* < 0.01 & *p* < 0.0001), further confirming the inhibitory effect of DUSP4 on myogenic differentiation **(**Fig. 2L-N**)**.

In summary, we have demonstrated that DUSP4 negatively regulates osteogenic and myogenic differentiation by inhibiting the p38 MAPK pathway. Conversely, miR-206-5p promotes both osteogenic and myogenic differentiation by targeting and suppressing DUSP4, thereby activating the p38 MAPK signaling pathway.

### Preparation and characterization of miR@DT/iMNV

**Fig. 3:**
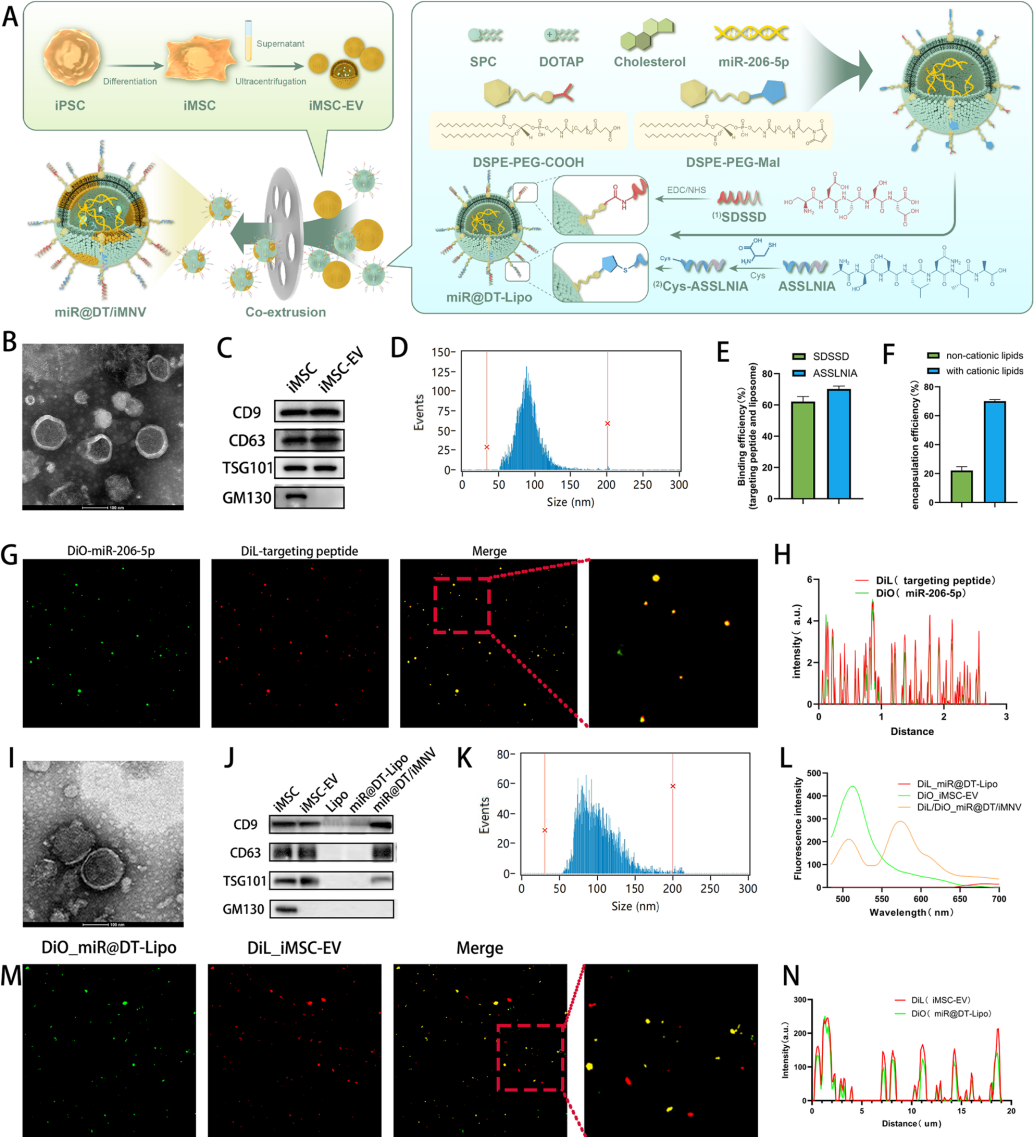
Preparation and characterization of miR@DT/iMNV. **(A)** Schematic diagram of the preparation of miR@DT/iMNV. **(B)** TEM image of iMSC-EV. **(C)** Western blot of specific makers of the iMSC and iMSC-EV. **(D)** Nanoflow cytometry detection of particle size distribution of iMSC-EVs. **(E)** Quantification of the binding efficiency of SDSSD and ASSLNIA (*n* = 3). **(F)** Quantification of the encapsulation efficiency of miR-206-5p (*n* = 3). **(G)** Fluorescence co-localization map of DiO_miR-206 and DiL_SDSSD/ASSLNIA. **(H)** Semi-quantification of fluorescence co-localization between DiO-miR-206-5p and DiL-SDSSD/ASSLNIA. **(I)** TEM image of miR@DT/iMNV. **(J)** Western blot of specific makers of the miR@DT/iMNV. **(K)** Nanoflow cytometry detection of particle size distribution of the miR@DT/iMNV. **(L)** FRET phenomenon of DiL_miR@DT-Lipo and DiO_iMSC-EV. **(M)** Fluorescence co-localization map of DiL_miR@DT-Lipo and DiO_iMSC-EV. **(N)** Semi-quantification of fluorescence co-localization between DiO_miR@DT-Lipo and DiL_iMSC-EV. Data are displayed as mean ± SD

Based on the above findings, we next designed and constructed a hybrid nanovesicle system (miR@DT/iMNV) for the efficient co-delivery of miR-206-5p to dual bone-muscle targets (preparation workflow in Fig. 3A). First, we induced iPSCs differentiation into iMSCs. Flow cytometry confirmed > 95% expression of positive markers (CD73, CD90, CD105) and negative markers (CD34, CD45, HLA-DR) (Figure S1**)**.

Next, iMSC-EVs were purified from culture supernatants via gradient ultracentrifugation. TEM showed cup-shaped lipid bilayer vesicles **(**Fig. 3B**)**. Western blot verified EV-specific markers (CD9, CD63, TSG101) but not Golgi-associated GM130 **(**Fig. 3C**)**. Nanoflow cytometry quantified a concentration of 7.50 × 10^9^ particles/mL and size of 90.82 nm **(**Fig. 3D**)** while zeta potential averaged − 9.38 mV (Fig. S2A).

For miR@DT-Lipo synthesis, we used DSPE-PEG-COOH (conjugated to bone-targeting peptide SDSSD) [40–42] and DSPE-PEG-Mal (conjugated to cys-linked muscle-targeting peptide ASSLNIA) [43, 44]. Positively charged DOTAP and miR-206-5p were incorporated to enhance loading efficiency [45]. Quantification of unreacted residues determined peptide binding efficiencies > 60% **(**Fig. 3E**)** and miR-206-5p encapsulation efficiency ≈ 70% **(**Fig. 3F**)**. Fluorescence co-localization (DiO-labeled miR-206-5p and DiL-labeled targeting peptides) validated synthesis **(**Fig. 3G-H**)**. We measured the average particle size of miR@DT-Lipo to be 118 nm, with a zeta potential of -10.37 mV (Figure S2B-C).

Finally, miR@DT/iMNV were formed by fusing iMSC-EVs and miR@DT-Lipo via co-extrusion [46]. TEM revealed retained cup-shaped morphology **(**Fig. 3I**)**, and Western blot showed EV markers (CD9, CD63, TSG101) **(**Fig. 3J**)**. Nanoflow cytometry indicated that the particle size of miR@DT/iMNV was larger than that of iMSC-EVs (100.73 nm vs. 90.82 nm; Fig. 3K), with zeta potential of -9.62 mV (Figure S2D). Fluorescence scanning demonstrated FRET in miR@DT/iMNV (Fig. 3L), while co-localization assays confirmed fusion efficiency **(**Fig. 3M-N**)**.

### Targeting and biocompatibility of miR@DT/iMNV

**Fig. 4:**
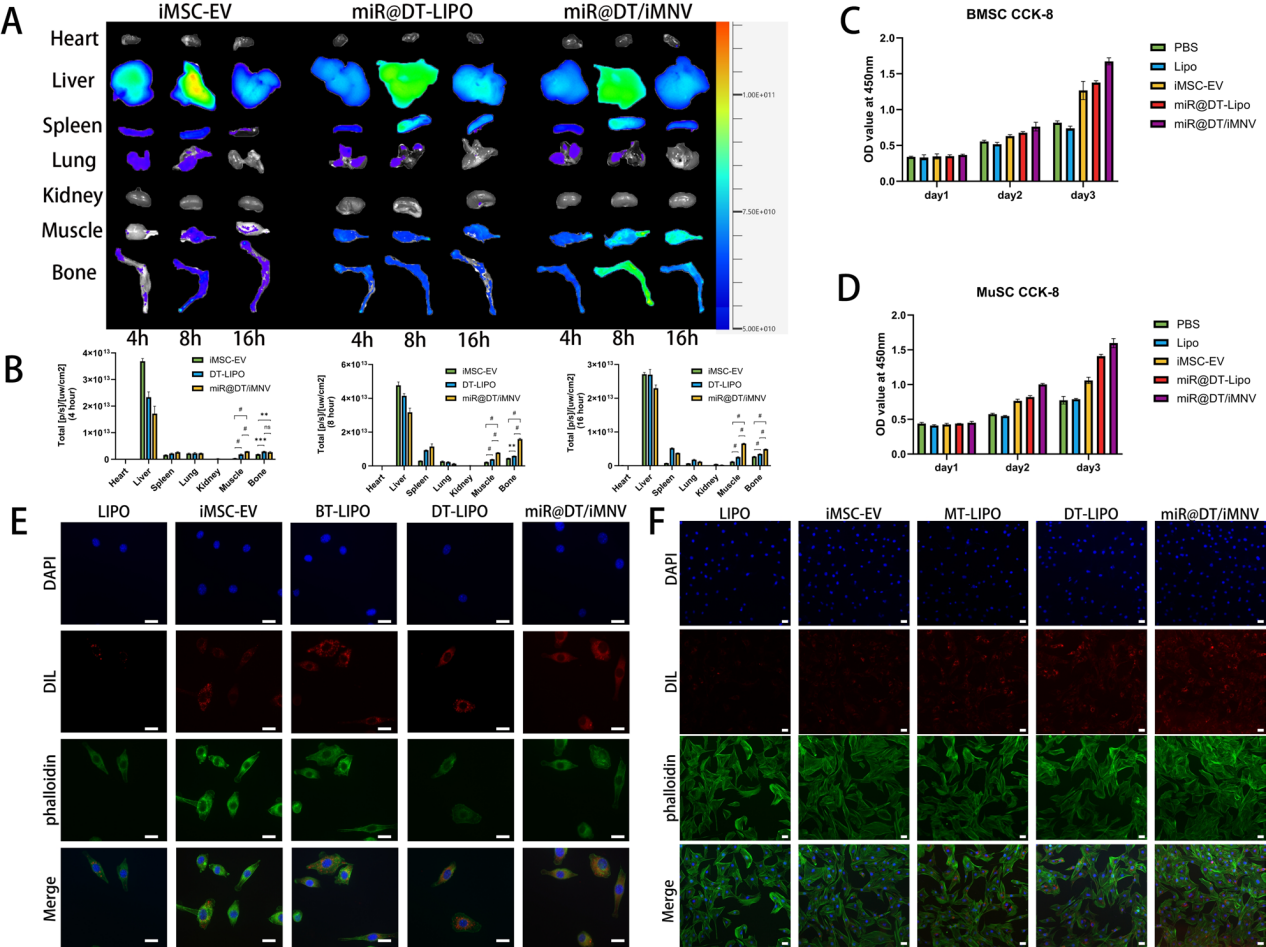
Targeting and biocompatibility of miR@DT/iMNV. **(A)** Biodistribution of DiR-labeled iMSC-EV, miR@DT-Lipo, or miR@DT/iMNV in mice. **(B)** Fluorescence intensity of DiR-labeled iMSC-EV, miR@DT-Lipo, or miR@DT/iMNV in different organs (*n* = 3). **(C**,** D)** CCK-8 cytotoxicity evaluation of miR@DT/iMNV on BMSCs and MuSCs (*n* = 3). **(E)** IF images of BMSCs uptake of DiL-labeled miR@DT/iMNV. **(F)** IF images of MuSCs uptake of DiL-labeled miR@DT/iMNV. *p < 0.05, **p < 0.01, ***p < 0.001, and #*p* < 0.0001. Scale bar: 20 μm. Data are displayed as mean ± SD

To further investigate the osteo-muscle dual-targeting capacity of miR@DT/iMNV, we performed biophotonic imaging in mice: after tail vein injection of DiR-labeled miR@DT/iMNV, we assessed its accumulation in bone and muscle tissues. Major organs—including visceral tissues, gastrocnemius (GAs), tibia, and femur—were collected for imaging analysis. The results showed that both bone and muscle tissues from mice in miR@DT-Lipo and miR@DT/iMNV groups exhibited significantly higher fluorescence signal intensity compared to those in the iMSC-EV group **(**Fig. 4A**)**. Quantitative analysis of fluorescence signals demonstrated that dual-targeting peptide modification significantly enhanced the enrichment of miR@DT/iMNV in bones and muscles while reducing distribution in non-target organs (at 16 h, *p* < 0.001) **(**Fig. 4B**)**. To illustrate the role of bone/muscle targeting peptides, we additionally engineered bone-targeted or muscle-targeted mono-liposomes. Fluorescence signals confirmed that such modifications enabled liposomes to accumulate preferentially in bones or muscles, respectively (Figure S3).

Subsequently, we verified the uptake ability of miR@DT/iMNV by BMSCs and MuSCs. First, we assessed the biocompatibility of miR@DT/iMNV in these cells. miR@DT/iMNV showed no cytotoxicity and even exhibited a certain proliferation-promoting effect **(**Fig. 4C-D**)**. Following a 2-hour incubation with DIL-labeled miR@DT/iMNV, we observed that both BMSCs and MuSCs internalized significantly more miR@DT-Lipo and miR@DT/iMNV than Lipo or iMSC-EV. Furthermore, bone-specific single-targeting liposomes were preferentially internalized by BMSCs, while muscle-specific ones were taken up by MuSCs—results that further confirm the dual-targeting capability of miR@DT/iMNV toward BMSCs and MuSCs **(**Fig. 4E-F**)**. Fluorescence quantitative analysis showed that after modification with targeting peptides, the efficiency of BMSCs in taking up miR@DT/iMNV was 3.8 times higher than that of Lipo and approximately 32% higher than that of iMSC-EV; the efficiency of MuSCs in taking up miR@DT/iMNV was about 3 times higher than that of Lipo and approximately 54% higher than that of iMSC-EV (Figure S4A-B). Given that MuSCs differentiate into myotubes during myogenesis, we specifically examined the targeting ability of miR@DT/iMNV toward myotubes. The results revealed that there was no significant difference in fluorescence distribution between MuSCs and myotubes (Figure S5).

### miR@DT/iMNV promotes osteogenic differentiation in vitro

One of the key mechanisms of miR@DT/iMNV in treating OSP lies in its ability to promote osteogenic differentiation. To validate this effect, we treated BMSCs with miR@DT/iMNV and observed changes in their osteogenic capacity.

**Fig. 5:**
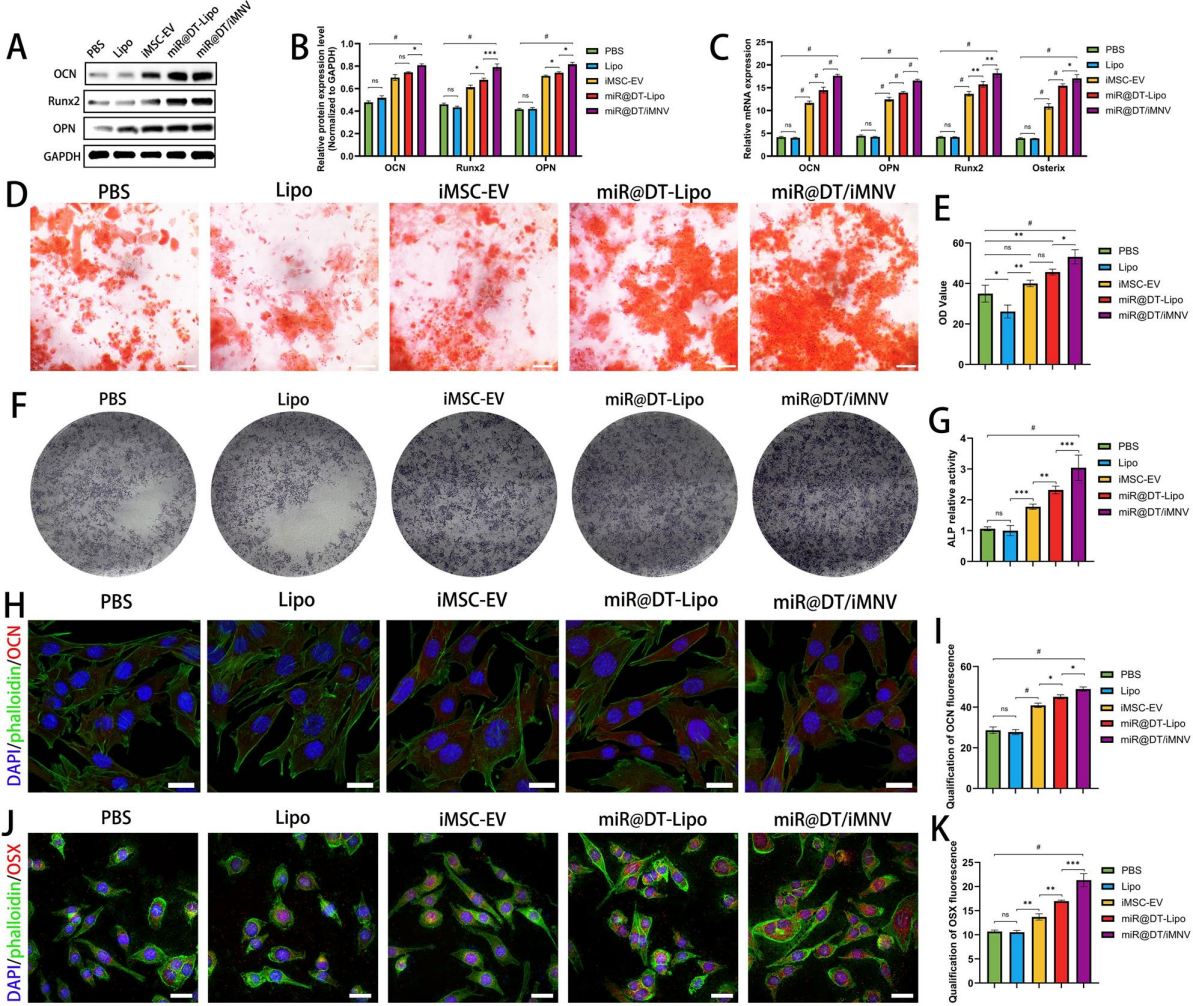
miR@DT/iMNV promotes osteogenic differentiation in vitro. (**A**) Western blot of osteogenesis-related protein (OCN, Runx2, OPN) after intervention with different nanoparticles for 7 days. (**B**) Quantitative analysis of osteogenesis-related protein (*n* = 3). (**C**) Relative mRNA expression of osteogenesis-related genes (OCN, OPN, Runx2 and OSX) after intervention with different nanoparticles for 7 days (*n* = 5). (**D**) Representative microscopic images showing mineralized matrix by ARS staining. (**E**) Quantitative analysis of ARS staining (*n* = 3). (**F**) Representative photographs of ALP staining during the osteogenic differentiation process. (**G**) ALP activity of BMSCs incubated with different nanoparticles for 14 days (*n* = 3). (**H**) Representative IF images showing expression of OCN by BMSCs after different treatments for 10 days. (**I**) Bar graph data showing the fluorescence intensity of OCN (*n* = 3). (**J**) Representative IF images showing expression of OSX by BMSCs after different treatments for 10 days. (**K**) Bar graph data showing the fluorescence intensity of OSX (*n* = 3). ^*^p < 0.05, ^**^p < 0.01, ^***^p < 0.001, and ^#^*p* < 0.0001. Scale bar: 20 μm. Data are displayed as mean ± SD

First, Western blot analysis revealed significantly increased OCN, Runt-related transcription factor 2 (Runx2), and osterix (OSX) protein levels in BMSCs following miR@DT/iMNV intervention **(**Fig. 5A-B**)**. Then, RT-qPCR confirmed miR@DT/iMNV-mediated upregulation of osteogenic genes (OCN, OPN, Runx2, and OSX) **(**Fig. 5C**)**. Alizarin Red staining demonstrated that after 21 days of osteogenic induction, the miR@DT/iMNV group exhibited a marked increase in mineralized nodules compared to the PBS group, surpassing both the iMSC-EV and miR@DT-Lipo groups. These results indicate that miR@DT/iMNV exhibited a more potent osteogenic-promoting effect compared to iMSC-EV and miR@DT-Lipo **(**Fig. 5D-E**)**. A similar trend was observed in ALP staining, which showed significantly enhanced ALP activity in the miR@DT/iMNV group **(**Fig. 5F-G**)**. Finally, immunofluorescence staining further assessed the expression of OCN and OSX across different groups. While iMSC-EV, miR@DT-Lipo, and miR@DT/iMNV all promoted the expression of these osteogenic markers, the miR@DT/iMNV group showed significantly higher than other groups **(**Fig. 5H-K**)**.

In summary, these findings demonstrate that miR@DT/iMNV robustly enhances osteogenic differentiation, suggesting its potential to achieve comparable therapeutic effects in vivo.

### miR@DT/iMNV facilitates in vitro myogenic differentiation via multiple mechanisms

**Fig. 6:**
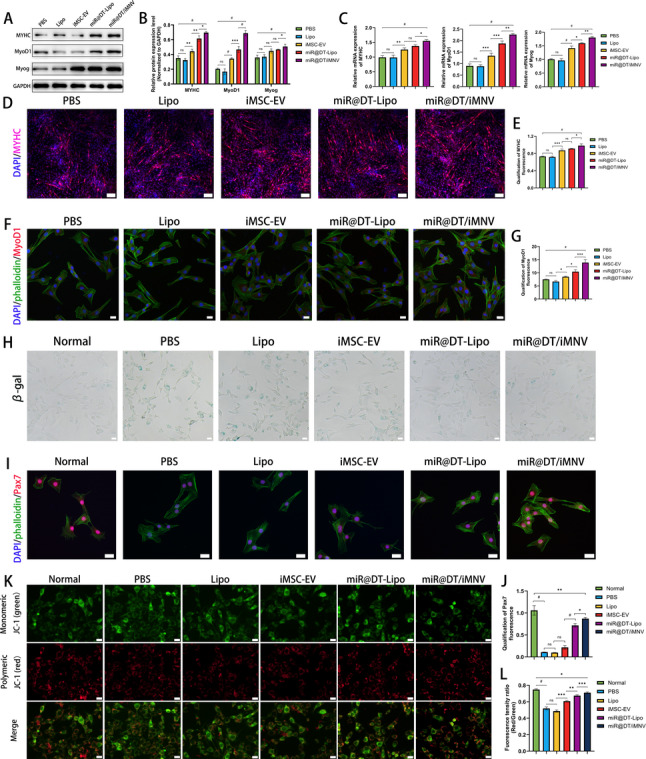
miR@DT/iMNV facilitates in vitro myogenic differentiation via multiple mechanisms. **(A)** Western blot of myogenesis-related protein (MYHC, MyoD1 and Myog) after intervention with different nanoparticles for 3 days. **(B)** Quantitative analysis of myogenesis-related protein (*n* = 3). **(C)** Relative mRNA expression of osteogenesis-related genes (MYHC, MyoD1 and Myog) after intervention with different nanoparticles for 3 days (*n* = 5). **(D)** Representative IF images showing expression of MYHC by MuSCs after different treatments for 3 days. Scale bar: 100 μm. **(E)** Bar graph data showing the fluorescence intensity of MYHC (*n* = 3). **(F)** Representative IF images showing expression of MyoD1 by MuSCs after different treatments for 3 days. Scale bar: 20 μm. **(G)** Bar graph data showing the fluorescence intensity of MyoD1 (*n* = 3). **(H)** Representative images of β-gal staining of normal MuSCs or senescence-induced MuSCs after different treatments. Scale bar: 20 μm. **(I)** Representative IF images showing expression of Pax7 by normal MuSCs or senescence-induced MuSCs after different treatments. Scale bar: 20 μm. **(J)** Bar graph data showing the fluorescence intensity of Pax7 (*n* = 3). **(K)** Representative JC-1 images showing ΔΨm of normal MuSCs or senescence-induced MuSCs after different treatments. **(L)** Quantitative analysis of fluorescence tensity ratio (Red/Green) (*n* = 3). Scale bar: 20 μm. ^*^p < 0.05, ^**^p < 0.01, ^***^p < 0.001, and ^#^*p* < 0.0001. Data are displayed as mean ± SD

After successfully validating that miR@DT/iMNV promotes osteogenic differentiation in vitro, we proceeded to investigate its role in modulating myogenic differentiation. First, we assessed the expression of myogenic regulatory factors (MYHC, MyoD1, and Myog) via Western blot **(**Fig. 6A-B**)**. The results demonstrated varying degrees of increased expression of these proteins under miR@DT/iMNV intervention. A similar trend was observed in RT-qPCR experiments, where the transcriptional activity of MYHC, MyoD1, and Myog was significantly enhanced following miR@DT/iMNV treatment (*p* < 0.0001) **(**Fig. 6C**)**. Subsequently, immunofluorescence staining revealed that during induced myogenic differentiation, miR@DT/iMNV intervention led to elevated expression of MYHC and MyoD1 **(**Fig. 6D-G**)**. Quantitative analysis confirmed that these changes were statistically significant compared to other groups (*p* < 0.0001).

Numerous studies, including our previous research, have indicated that the decline in myogenic differentiation capacity in sarcopenia may be associated with impaired mitochondrial function [47–50]. Mechanistically, the p38 MAPK pathway is an important participant in mitochondrial metabolism [20]. Meanwhile, GO analysis of miR-206-5p also highlighted its strong correlation with mitochondria in terms of cellular components, suggesting that improving mitochondrial function could serve as a therapeutic target for enhancing myogenic differentiation. Given that aging is a well-known contributor to mitochondrial dysfunction, we first established an oxidative stress-cellular senescence-mitochondrial dysfunction cell model by treating MuSCs with hydrogen peroxide [51]. In β-galactosidase staining experiments, we observed significantly deeper staining in the hydrogen peroxide-treated group compared to the normal cell group, whereas miR@DT/iMNV intervention reversed the degree of senescence **(**Fig. 6H**)**. Additionally, we assessed the expression of Pax7 protein via immunofluorescence staining. Pax7 is a marker molecule that maintains the stemness of MuSCs [52]. In this oxidative stress-cellular senescence-mitochondrial dysfunction model, a significant decrease in Pax7 expression was observed, indicating impaired stemness. Following miR@DT/iMNV intervention, this phenomenon was notably ameliorated (*p* < 0.0001). Quantitative immunofluorescence analysis revealed that Pax7 expression levels were restored to nearly normal levels, demonstrating that miR@DT/iMNV plays a crucial role in recovering MuSCs stemness **(**Fig. 6I-J**)**. Finally, we assessed mitochondrial membrane potential (ΔΨm) using the JC-1 assay. As shown, normal satellite cells exhibited high membrane potential, whereas hydrogen peroxide-induced modeling caused significant mitochondrial damage, characterized by low membrane potential. This damage was markedly ameliorated by miR@DT/iMNV treatment; quantitative analysis revealed a significant increase in the red/green fluorescence intensity ratio, indicative of substantial recovery of mitochondrial function **(**Fig. 6K-L**)**.

In summary, miR@DT/iMNV significantly enhances the myogenic differentiation of satellite cells while exerting anti-senescence effects, maintaining satellite cell stemness, and improving mitochondrial function. These mechanisms collectively contribute to the myogenic differentiation process, serving as critical factors in promoting in vitro myogenic differentiation.

### miR@DT/iMNV mediates the polarization of macrophages from M1 to M2 phenotype

Previous studies have demonstrated that chronic inflammation serves as a critical pathological factor in the development and progression of both sarcopenia and osteoporosis [53–57]. Our earlier functional predictions of miR-206-5p implicated numerous inflammation-related functions and pathways. In addition, previous studies have also found that MSC-EVs possess certain immunomodulatory functions. In a 2020 study, Kim et al. demonstrated through both in vitro and in vivo experiments that MSC-EVs exhibit significant immunosuppressive capabilities. These vesicles are enriched with key molecules such as TGF-β1, PTX3, let-7b-5p, and miR-21-5p, which directly inhibit the TCR/TLR4 signaling pathway and reduce the secretion of pro-inflammatory cytokines [58]. Similarly, in a recent review, Zhang et al. revealed from an epigenetic perspective that MSC-EVs can reprogram the epigenetic state of recipient cells by delivering non-coding RNAs, thereby promoting macrophage M2 polarization, Treg cell differentiation, and inflammation alleviation to achieve long-term immune regulation [59]. We therefore hypothesized that hybrid vesicles might modulate inflammatory cell phenotypes, shifting them from pro-inflammatory to anti-inflammatory states, thereby promoting osteogenic and myogenic differentiation processes.

**Fig. 7:**
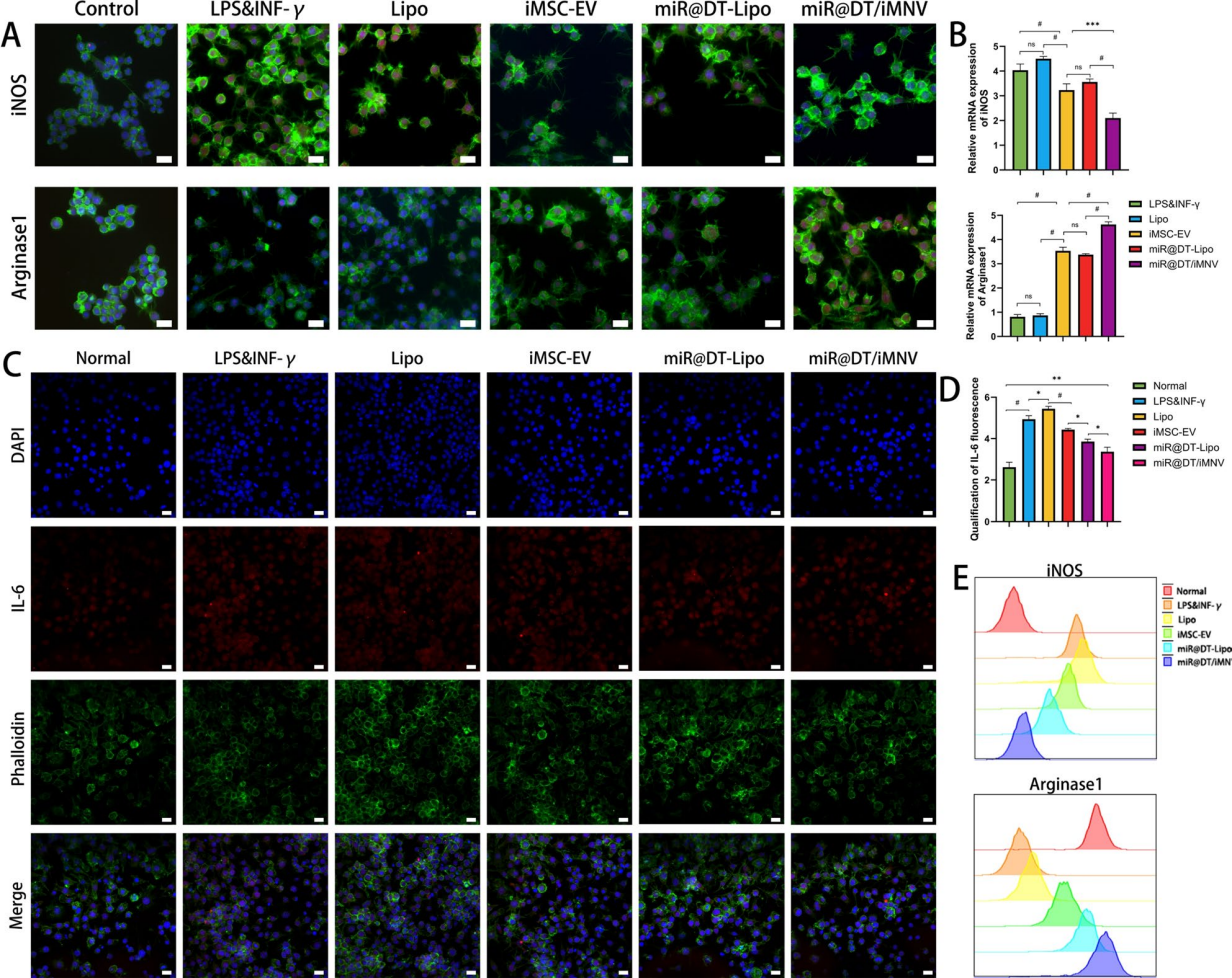
miR@DT/iMNV alters the M1 phenotype of inflammatory cells. **(A)** Immunofluorescence images of iNOS and Arginase 1 in RAW264.7 cells under normal conditions, after M1 polarization induced by LPS & INF-γ, and after intervention with Lipo, iMSC-EV, miR@DT-Lipo or miR@DT/iMNV following polarization. **(B)** Relative mRNA expression levels of iNOS and Arginase 1 in macrophages under different intervention conditions (*n* = 5). **(C)** IF images of IL-6 macrophages after the treatment in (A). **(D)** Quantification of the immunofluorescence intensity of IL-6 (*n* = 3). **(E)** FCM analysis of iNOS and Arginase1 in macrophages under different intervention conditions. ^*^p < 0.05, ^**^p < 0.01, ^***^p < 0.001, and ^#^*p* < 0.0001. Scale bar: 20 μm. Data are displayed as mean ± SD

To investigate the role of miR@DT/iMNV in chronic inflammation, we first established an in vitro inflammatory cell model by inducing M1 polarization in RAW264.7 macrophages. Subsequently, we treated the cells with Lipo, iMSC-EV, miR@DT-Lipo, and miR@DT/iMNV, respectively. Immunofluorescence results showed that untreated RAW264.7 cells displayed spherical or ovoid morphology, whereas LPS&INF-γ-induced cells showed characteristic M1 morphology: flattened soma, extensive filopodia, and dispersed distribution, confirming successful M1 polarization. Following intervention, the iMSC-EV, miR@DT-Lipo, and miR@DT/iMNV groups-all demonstrated a trend of decreased secretion of the pro-inflammatory marker iNOS and increased expression of the anti-inflammatory marker Arginase1, with miR@DT/iMNV exhibiting the strongest effect, suggesting its superior anti-inflammatory efficacy **(**Fig. 7A**)**. RT-qPCR also showed that miR@DT/iMNV exhibits significant anti-inflammatory effects **(**Fig. 7B**)**. We further measured the expression of the pro-inflammatory cytokine IL-6 across groups. As shown in Fig. 7C, miR@DT/iMNV intervention reduced pro-inflammatory cytokine expression, confirming its anti-inflammatory effect. Quantitative analysis of IL-6 immunofluorescence intensity further validated this finding **(**Fig. 7D**)**. Additionally, Flow cytometry yielded consistent results, demonstrating that miR@DT/iMNV treatment mediated the polarization of macrophages from the M1 to the M2 phenotype **(**Fig. 7E**)**.

In summary, our experiments confirm that miR@DT/iMNV can reprogram inflammatory cell phenotypes, shifting them from pro-inflammatory to anti-inflammatory states, which may represent one of the therapeutic targets of miR@DT/iMNV in treating OSP.

### miR@DT/iMNV improves OSP in mice

**Fig. 8:**
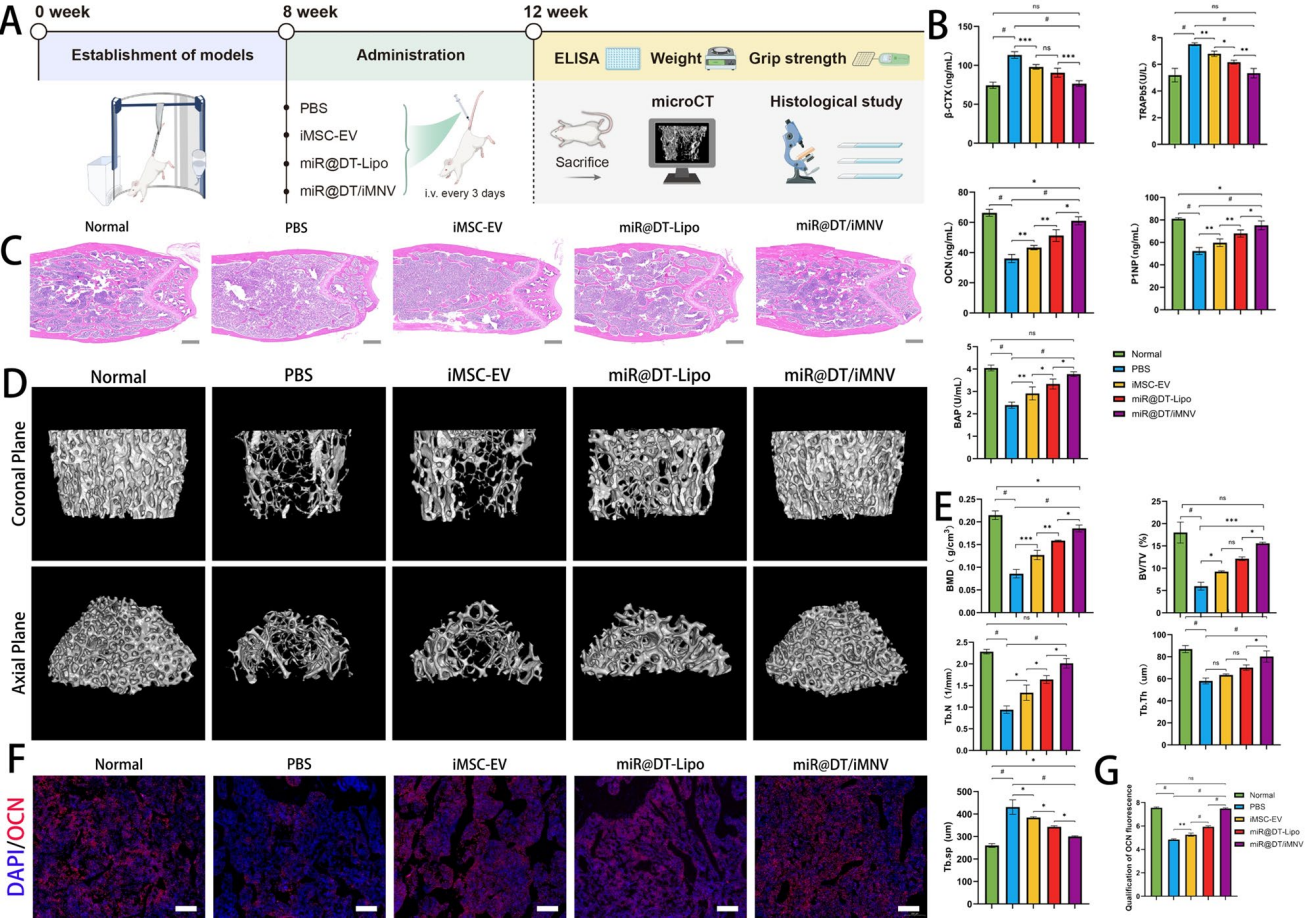
miR@DT/iMNV improves osteogenic function in OSP mice. (**A**) Schematic diagram of OSP model construction and animal experimental procedure. (**B**) Quantitative analysis of the concentrations of bone metabolism biochemical indicators in the serum including BAP, BGP, TRAP5b, β-CTX, and P1NP (*n* = 5). (**C**) H&E staining of the distal femurs. Scale bar: 500 μm. (**D**) Representative three-dimensional reconstructed micro-CT images showing the trabeculae in distal femurs. (**E**) Quantitation of BMD, BV/TV, Tb. N, Tb. Th, and Tb. Sp (*n* = 3). (**F**) Representative IF images showing expression of OCN after different treatments. Scale bar: 200 μm. (**G**) Bar graph data showing the fluorescence intensity of OCN. ^*^p < 0.05, ^**^p < 0.01, ^***^p < 0.001, and ^#^*p* < 0.0001. Data are displayed as mean ± SD

Following the demonstration that miR@DT/iMNV exerts potent osteogenic and myogenic promotional effects, we further evaluated that in vivo therapeutic efficacy. Figure 8A schematically depicts our strategy for establishing the OSP mouse model via tail suspension along with the design of the overall animal experiments [60–62]. 

#### miR@DT/iMNV exhibits the ability to restore bone mass in mice

After the completion of the 8-week modeling period and 4-week treatment period, we first performed the ELISA assays. Results showed elevated levels of osteogenic markers and decreased levels of osteoclastic markers **(**Fig. 8B**)**. Consistently, Hematoxylin-eosin (H&E) staining further confirmed that miR@DT/iMNV treatment induced extensive new bone formation, organized lamellar structure with reduced osteolytic lacunae compared to other control groups **(**Fig. 8C**)**. For the assessment of bone mass recovery, reconstructed 3D micro-CT images revealed that after tail vein injection of the therapeutic agent, OSP mice in the miR@DT/iMNV group exhibited a significant increase in bone volume, with bone morphology approaching that of normal mice. A moderate degree of bone mass recovery was also observed in the iMSC-EV and miR@DT-Lipo groups **(**Fig. 8D**)**. This trend was clear across various bone parameters, including bone mineral density (BMD), bone volume fraction (BV/TV), trabecular thickness (Tb. Th) and so on **(**Fig. 8E**)**. Finally, immunofluorescence staining of bone tissue paraffin sections revealed that OCN expression in the miR@DT/iMNV group was significantly higher than in other control groups (*p* < 0.0001) **(**Fig. 8F-G**)**. In summary, miR@DT/iMNV treatment significantly increased bone mass in OSP mice and markedly ameliorated their osteoporotic condition.

#### miR@DT/iMNV exhibits the capacity to restore muscle mass

**Fig. 9:**
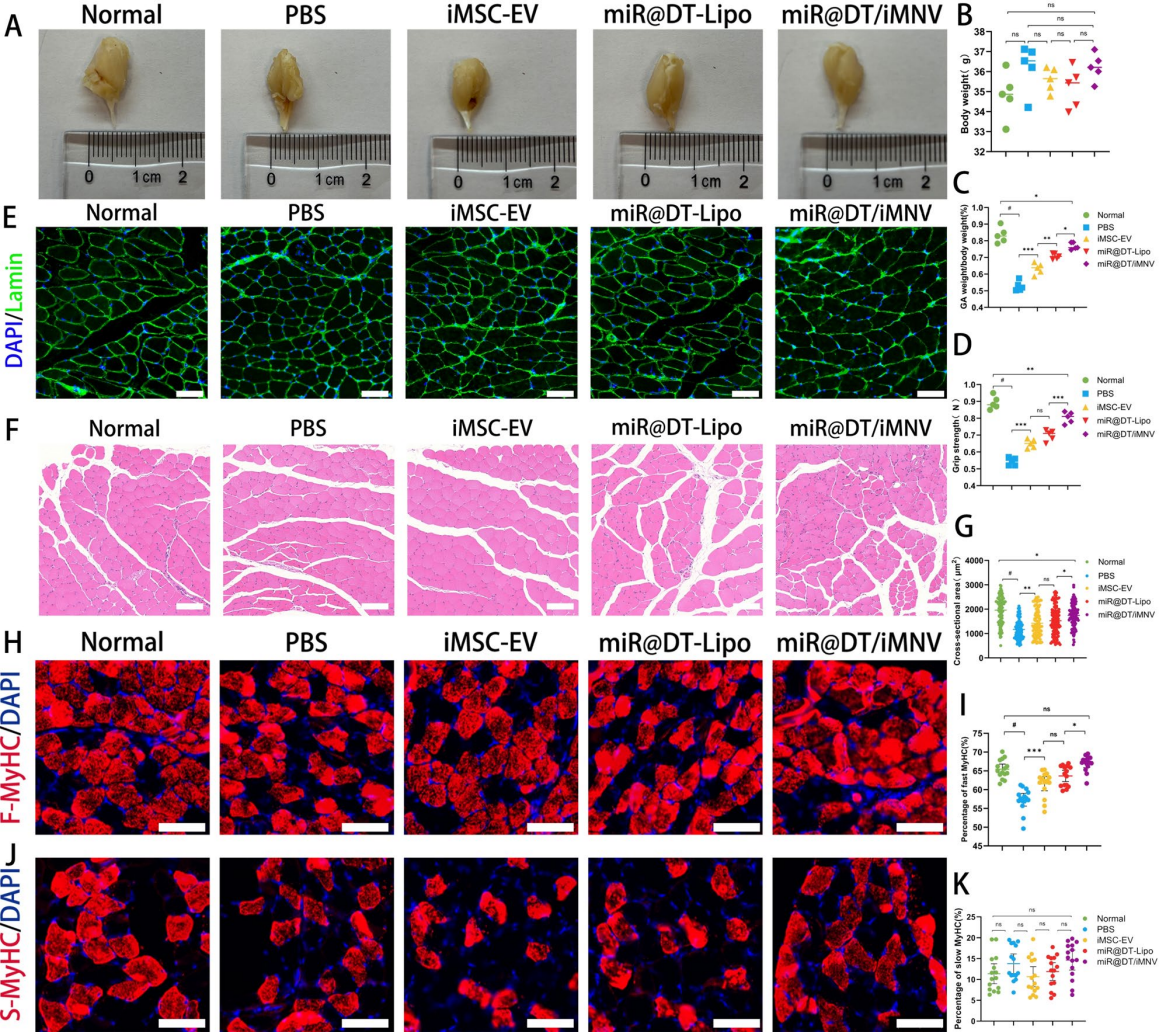
miR@DT/iMNV improves myogenic function in OSP mice. **(A)** Representative GAs macro photographs. **(B)** Quantitative analysis of body weight of mice in different groups (*n* = 5). **(C)** Quantitative analysis of GAs weight normalized to body weight of mice in different groups (*n* = 5). **(D)** Quantitative analysis of grip strength of mice in different groups (*n* = 5). **(E)** Representative IF staining of Laminin. **(F)** Representative H&E staining. **(G)** Quantitative analysis of CSA (*n* = 15). **(H)** Representative IF staining of fast MyHC. **(I)** Quantitative analysis of the percentage of fast MyHC (*n* = 15). **(J)** Representative IF staining of slow MyHC. **(K)** Quantitative analysis of the percentage of slow MyHC (*n* = 15). Scale bar: 200 μm. ^*^p < 0.05, ^**^p < 0.01, ^***^p < 0.00, and ^#^*p* < 0.0001. Data are displayed as mean ± SD

Similarly, we designed a series of experiments to observe the muscle status of OSP mice after treatment with miR@DT/iMNV. First, we conducted a preliminary assessment of the motor ability, muscle function, and general physiological status of the mice. Specifically, this included treadmill endurance tests, pole-climbing behavior tests, as well as systematic measurements of body weight and hindlimb muscle strength. After the mice were sacrificed, their GAs were collected, weighed, and measured. All data were collated and subjected to quantitative analysis. In the treadmill running test, OSP mice exhibited a reduction in both running duration and total distance, indicating impaired exercise endurance. Following treatment, this decline was partially reversed, with the miR@DT/iMNV group showing the most significant restoration of performance (Figure S6A-B). During the pole-climbing test, OSP mice demonstrated a higher frequency of slips and falls and required significantly more time to complete the task compared to normal controls. Similarly, administration of miR@DT/iMNV led to observable improvement in these motor coordination deficits (Figure S6C-D). As shown in Fig. 9A, the GAs morphology of mice in the miR@DT/iMNV group was more robust than that in the other groups and was close to the muscle state of normal mice. The results showed no significant differences in body weight among the different treatment groups (*p* > 0.05) **(**Fig. 9B**)**. However, the ratio of GAs weight to body weight revealed markedly different outcomes: OSP mice exhibited a lower ratio, which increased significantly after miR@DT/iMNV treatment (*p* < 0.0001) **(**Fig. 9C**)**. Hindlimb grip strength followed the same trend, demonstrating that miR@DT/iMNV treatment significantly improved muscle quality in OSP mice **(**Fig. 9D**)**. Subsequently, muscle sections were subjected to HE staining and immunofluorescence staining to calculate the cross-sectional area (CSA) of muscle fibers. The results indicated varying degrees of CSA increase in the iMSC-EV, miR@DT-Lipo, and miR@DT/iMNV treatment groups, with miR@DT/iMNV showing superior therapeutic effects compared to the other two groups and a significant improvement over OSP mice (*p* < 0.0001) **(**Fig. 9E-G**)**. Furthermore, immunofluorescence staining and quantitative analysis of fast and slow myosin in the GAs revealed a significant increase in fast myosin expression in the miR@DT/iMNV treatment group (*p* < 0.0001), while slow myosin expression showed no notable differences across groups (*p* > 0.05) **(**Fig. 9H-K**)**. This discrepancy may stem from their distinct roles in energy metabolism and physiological function [63, 64]. Additionally, we evaluated the biosafety of miR@DT/iMNV injection and found no significant toxic effects on major organs in mice (Figure S7).

Based on these experimental results, we conclude that the hybrid vesicles significantly enhance myogenic capacity in OSP mice and exhibit a pronounced therapeutic effect on the decline in muscle quality.

## Conclusion

In this study, we developed a dual-targeting hybrid nanovesicle (miR@DT/iMNV) based on the fusion of iPSC-MSC-derived exosomes and engineered liposomes, aiming to address the bottleneck in nucleic acid drug delivery for the treatment of OSP. The miR@DT/iMNV not only enabled the precise delivery of miR-206-5p to bone and muscle tissues but also effectively blocked the DUSP4/p38 MAPK negative feedback loop, thereby restoring impaired osteogenic and myogenic differentiation capacity. Compared with existing single delivery systems, our hybrid nanovesicle strategy demonstrated significant advantages in biocompatibility, targeting efficiency, and translational potential.

Efficient delivery of nucleic acid drugs remains a core challenge in regenerative medicine. Polymeric nanoparticles (PNPs), such as those based on PLGA or PEI, have been widely used in gene therapy, but their limitations cannot be overlooked. To achieve efficient nucleic acid compaction and endosomal escape, PNPs typically require high-density surface cationic charges, which often lead to nonspecific cell membrane disruption, cytotoxicity, and activation of inflammatory responses [65]. In the pathological environment of OSP, which is accompanied by chronic low-grade inflammation, introducing carriers with potential pro-inflammatory risks may counteract therapeutic benefits. Moreover, synthetic polymers lack intrinsic tissue homing receptors; relying solely on the EPR effect or chemically conjugated ligands often fails to overcome biological barriers in vivo, such as protein corona formation [66].

Although lipid nanoparticles (LNPs) are currently a mainstream choice for nucleic acid delivery, they face unique challenges in systemic therapy [67]. First, LNPs exhibit strong liver tropism after intravenous injection, primarily due to ApoE-mediated hepatocyte uptake, which significantly limits their bioavailability in extrahepatic tissues such as bone and muscle [68]. Second, the polyethylene glycol (PEG) modification introduced to maintain colloidal stability can, upon repeated administration, induce anti-PEG antibodies, leading to the “accelerated blood clearance (ABC)” phenomenon [69] and thereby compromising long-term efficacy [70].

In contrast, our miR@DT/iMNV integrates the dual advantages of natural EVs and synthetic liposomes. By incorporating membrane components from iMSC-EVs, the hybrid vesicles inherit the immune-privileged surface of EVs [71], granting the carrier a “self-cell”-like stealth capability that reduces clearance by the mononuclear phagocyte system and diminishes reliance on exogenous polymers. Simultaneously, the liposomal component enables high-efficiency encapsulation of miR-206-5p (overcoming the low drug-loading capacity of natural EVs) and, via pre-assembly technology, stably incorporates dual-targeting peptides (SDSSD and ASSLNIA), achieving more precise tissue specificity than natural EVs. This bio-synthetic synergy fundamentally overcomes the toxicity/immunogenicity issues of synthetic carriers and the engineering challenges of natural carriers [72].

The pathological mechanisms of OSP are complex, involving stem cell depletion, inflammatory microenvironment, and mitochondrial dysfunction. The hybrid nanovesicles in this study are not merely nucleic acid carriers but are inherently bioactive. Unlike PLGA or lipid carriers, the iMSC-EV component retains various anti-inflammatory factors, metabolic enzymes, and regenerative proteins secreted by the parental cells [73]. Our in vitro experiments confirmed that iMSC-EVs alone could promote macrophage polarization from the M1 to the M2 phenotype and improve mitochondrial membrane potential. This indicates that miR@DT/iMNV exerts a synergistic therapeutic effect: the vehicle components ameliorate the inflammatory microenvironment, paving the way for tissue regeneration, while miR-206-5p precisely reactivates the p38 MAPK signaling pathway to drive differentiation of parenchymal cells. This multi-target, multi-mechanism intervention model is well-suited for multifactorial syndromes such as OSP.

Despite the encouraging results, transitioning from a laboratory prototype to clinical application requires addressing challenges in scalability and manufacturing. First, standardization of cell sources is critical. Primary MSCs suffer from donor heterogeneity and limited expansion capacity, making it difficult to meet the demands for clinical-grade EV production. This study utilized iPSC-derived MSCs as production cell factories, theoretically providing an unlimited and genetically consistent starting material, which forms the basis for batch-to-batch consistency. However, large-scale expansion of iMSCs requires shifting from traditional 2D culture to GMP-compliant 3D bioreactor systems (e.g., microcarrier-based suspension culture), necessitating strict control of fluid shear stress to maintain EV yield and phenotypic stability [74–75]. Second, EV isolation and purification must move beyond reliance on ultracentrifugation. Ultracentrifugation is not linearly scalable and can cause vesicle aggregation and damage. Future clinical manufacturing processes should adopt tangential flow filtration (TFF) coupled with size exclusion chromatography (SEC). TFF offers high throughput, low shear force, and scalability, efficiently removing free proteins and concentrating vesicles, making it the gold standard for industrial EV downstream processing [76]. Finally, the preparation process for hybrid nanovesicles requires scale-up optimization. The membrane extrusion method used in this study performed well for small-scale preparation but faces challenges of membrane clogging and pressure control during industrial-scale production. Microfluidic mixing technology represents a future solution. By precisely controlling the laminar mixing of liposomes and EVs in microchannels, microfluidics enables highly reproducible fusion, producing clinical-grade hybrid nanomedicines with uniform particle size (PDI < 0.1) and consistent drug loading [77].

This study has certain limitations. First, the tail-suspension model used primarily simulates disuse-induced OSP and does not fully replicate the complex hormonal changes or systemic aging characteristics of OSP patients. Future studies should validate efficacy in natural aging or genetic knockout models. Second, as a complex bio-synthetic hybrid, the heterogeneity of hybrid nanovesicle components presents significant characterization challenges, necessitating the finer quality control standards. Moreover, although short-term biocompatibility is favorable, whether long-term repeated injections of hybrid nanovesicles containing synthetic peptides could induce adaptive immune responses requires further long-term toxicological evaluation.

In summary, miR@DT/iMNV represents a versatile and highly translatable nanomedicine platform. By addressing the efficiency-safety dilemma in nucleic acid delivery and integrating anti-inflammatory and pro-regenerative mechanisms, this strategy offers new hope for the treatment of OSP.

## Methods

### Preparation of miR@DT-Lipo

Liposomes were prepared using the thin-film hydration method. A mixture of SPC: Chol: DOTAP: DSPE-PEG-COOH: DSPE-PEG-Mal = 50: 40: 8: 1: 1 was dissolved in a chloroform: methanol (2:1) solvent system, with a total lipid concentration of 5 mM. The solution was then transferred to a round-bottom flask and evaporated at 55 °C for 30 min using a rotary evaporator to form a uniform lipid film, followed by vacuum drying for 2 h to remove residual solvents. The film was hydrated with a citrate buffer containing miR-206-5p (20 µg/mL) at 55 °C for 30 min with intermittent vortexing. Sonication (50 W, 3 × 10-second pulses) was applied to enhance encapsulation efficiency. The liposomes were extruded 5–7 times through a 100 nm polycarbonate membrane and purified using a Sepharose CL-4B column to remove unencapsulated miR-206-5p.

The bone-targeting peptide SDSSD and muscle-targeting peptide ASSLNIA (purity ≥ 98% as determined by HPLC) were synthesized by Wanshenghaotian Biotechnology (Shanghai, China) and dissolved in a 5 mM tris(2-carboxyethyl) phosphine hydrochloride (TCEP) solution at a concentration of 1 mg/ml. The carboxyl groups on the liposomes were first activated with EDC/NHS, followed by the addition of SDSSD. The mixture was stirred at 37 °C in the dark for 2 h. Afterward, ultracentrifugation (100,000×g, 30 min) was performed, and the supernatant was discarded. The pellet was resuspended in PBS and washed twice to remove unreacted components.

For the muscle-targeting peptide ASSLNIA, it was first reacted with cysteine (cys) to introduce a thiol group, yielding cys-ASSLNIA. The cys-ASSLNIA was then added to the liposome system, and the pH was adjusted to 6.5–7.0 with PBS. The mixture was stirred at 37 °C in the dark for 1 h. Another round of ultracentrifugation was conducted to obtain the engineered liposomes, designated as miR@DT-Lipo.

### Derivation of iMSCs from iPSCs

Human iPSCs (cell line DYR0100) were routinely grown and proliferated on ESC-Qualified Matrigel (BD Biosciences, Franklin Lakes, NJ, USA) in mTESR1 (StemCell Technologies, Vancouver, Canada) in sixwell plates. When the cells were 80% confluent, the mTESR1 culture system was exchanged for an MSC differentiation medium kit (Nuwacell, China). After culture for 14 days in differentiation medium according to the protocols of the kit, the cells were digested with 0.25%trypsin-EDTA (Gibco/Life Technologies, Carlsbad, CA, USA) and then reseeded in 0.1% gelatine-coated 25-cm^2^ culture flasks at a density of 5 × 10^4^/mL in MSC culture medium (Nuwacell, Hefei, China). The cells were named Passage 1 upon reaching confluence. Generally, at Passage 3, they displayed a typical fibroblast-like morphology and were then used to analyze surface markers, differentiation capacity and for further experiments.

### EVs isolation

EVs were isolated from cell culture supernatant as described previously [78]. Briefly, iMSCs were cultured in serum-free MSC medium for 48 h. Then the conditioned medium was harvested and centrifuged at 300×g for 10 min to remove non-adherent cells, then at 2000×g for 20 min to remove dead cells and debris, and finally at 10^4^×g for 30 min to remove large vesicles. Then the supernatant was transferred to an ultracentrifuge (Beckman Coulter, Brea, CA, US) and centrifuged at 10^5^×g for 75 min. The cell pellet was resuspended with PBS and centrifuged at 10^5^×g for another 75 min to remove contaminating proteins. All these steps were conducted at 4 ℃.

### Exosome and miR@DT/iMNV characterization

The microstructure was observed and imaged by a transmission electron microscope (talos120, FEI, USA) at 120 kV. The size distribution and particle concentration were measured by a NanoFCM (U30, NanoFCM Inc, Xiamen, China). The zeta potential was measured by Zetasizer Nano ZS90 (Malvern, UK).

### Fluorescence co-localization assay

First, miR-206-5p was labeled with DiO and muscle/bone-targeting peptides were labeled with DiI to observe their co-localization under fluorescence microscopy. Semi-quantitative analysis of co-localization degree was performed to confirm the successful synthesis of miR@DT-Lipo. Subsequently, miR@DT-Lipo was labeled with DiO and iMSC-EV was labeled with DiI to observe their co-localization under fluorescence microscopy. Semi-quantitative analysis of co-localization degree was then conducted to verify the successful synthesis of miR@DT/iMNV.

### Verification of miR@DT/iMNV fusion

To assess the fusion efficiency of miR@DT-Lipo and iMSC-EV, DiL-labeled miR@DT-Lipo and DiO-labeled iMSC-EV were employed to create co-labeled miR@DT/iMNV during the fusion process. The incorporation of fluorescently labeled miR@DT-Lipo and iMSC-EV was visualized using a Full Spectrum Ultra High Resolution Laser Confocal Scanning System (Stellaris STED, Leica, Germany). Additionally, FRET technology was utilized to investigate the fusion of membranes [79–81]. In brief, miR@DT/iMNV were labeled with DiI (λex/λem = 550/567nm) and DiO (λex/λem = 484/501nm). Subsequently, the hybrid dye labeled miR@DT/iMNV were examined at λex=460 nm, and the fluorescence spectra were recorded ranging from 485 to 700 nm.

### Cell Isolation and culture

To isolate BMSCs and MuSCs, the 4-week-old mice were sacrificed and disinfected using 75% ethyl alcohol. For BMSCs isolation, intact marrow plugs rinsed and digested with enzymatic buffer (4 g/L dispase, 3 g/L type I collagenase, and 1 U/mL DNase I in HBSS buffer with calcium and magnesium) for 15 min at 37 ℃ to obtain a single-cell suspension. Subsequently, the enzymatic reaction was stopped by HBSS with 2 mM EDTA and 2% FBS. For MuSCs isolation, hindlimb skeletal muscles, including GAs, tibialis anterior muscle (TA), and quadriceps femoris, were dissected. The muscle tissue was minced into a smooth pulp and digested with 0.2% collagenase type II (C6885, Sigma, USA) for 2 h at 37 °C. Next, the homogenate was filtered using 70 μm nylon mesh cell strainer (431 751, Corning, USA). Centrifuge the homogenate at 1500 rpm for 10 min and resuspend the pellet gently in 10 mL of growth medium. The solution was added to a dish and incubated at 37 °C in 5% CO^2^ for 2 h. Then the supernatant contenting SCs was added to a new dish to separate suspending SCs and adherent fibroblasts. These cells were cultured in α minimum essential medium (α-MEM) supplemented with 20% FBS, 100 µg/mL streptomycin, and 100 U/mL penicillin at 37 ℃.

The murine RAW264.7 macrophage cells (Chinese Academy of Sciences, Shanghai, China) were cultured in DMEM supplemented with 10% FBS (V/V) at 37 ℃ in 5% CO^2^ humidified air. The RAW264.7 cells were treated with 100ng/mL LPS and 20ng/mL IFN-γ for 24 h in to induce the M1 phenotype.

### Osteogenic induction and evaluation

BMSCs were reseeded into 12-well plates at a density of 2 × 10^4^ cells per well. After 24 h, the medium was exchanged for α-MEM supplemented with osteogenic induction factors (10 nM dexamethasone, 5 mM β-glycerophosphate and 50 µg/mL ascorbic acid). In all cultures the medium was changed every 3 days. Alizarin red staining (ARS) and Alkaline Phosphatase (ALP) activity assay were used to assess the osteogenic effect. For the ALP activity assay, the cells were treated for 7 days. Briefly, the cells were washed gently four times using PBS and fixed in 4% paraformaldehyde for 15 min, then evaluated using an ALP assay kit (Beyotime) in accordance with the manufacturer’s protocol. For ARS, the cells were treated for 14 days. After washing and fixing as above, the cells were stained with 2% Alizarin red (Sigma) for 15 min and washed gently with PBS. Each plate was observed and photographed under a fluorescence microscope. For quantitative analysis, 10% acetic acid was added to the cells. After incubation for 12 h, the mixture of cells and acetic acid was centrifuged for 10 min at 18,000×g, then the supernatant was removed and neutralized with 10% ammonium hydroxide. Finally, 100 µL of each sample was removed to a well of a 96-well plate and the absorbance was measured at 405 nm using a microplate reader.

### Myogenic differentiation

The MuSCs were seeded in 12-well plates. After 80–90% confluence, the growth medium was replaced by differentiation medium (high glucose DMEM medium supplemented with 2% horse serum and 1% penicillin/streptomycin). Myogenic differentiation assay was performed for 7 days.

### qRT-PCR

Cells were harvested and treated with Trizol reagent, and total RNA was obtained according to the supplier’s protocols. A NanoDrop-2000 spectrophotometer (Thermo Fisher Scientific) was used to determine the concentration of RNA. Then miRNA samples were reverse transcribed using a stem-loop reverse transcriptase primer kit (Ribobio, Guangzhou, China). qRT-PCR was conducted with a SYBR Prime Script kit (Takara Bio Inc., Shiga, Japan).

### Western blot analysis

A protein extraction kit (Beyotime) was used to isolate the total proteins from harvested cells in accordance with the protocols provided by the manufacturer. Equal amounts of protein sample were loaded and separated by SDS-PAGE, then transferred to 0.22-µm polyvinylidene difluoride (PVDF) membranes. The membranes were blocked with 5% skimmed milk and incubated using specific antibodies overnight. The primary antibodies used were: CD9 (1:1000), CD63 (1:1000), TSG101 (1:1000), GM130 (1:1000), GAPDH (1:1000) (all from Abcam); OCN (1:1000), Runx2 (1:1000), OPN (1:1000), Myod1 (1:1000), eMyHC (1:1000), Myog (1:1000), DUSP4 (1:1000), p38 (1:1000) ,p-p38 (1:1000) (all from Servicebio). Finally, we incubated the membranes with horseradish peroxidase (HRP)-labelled secondary antibodies for 1 h at room temperature.

### Dual-luciferase reporter assay

A dual luciferase reporter gene assaykit (KEG3308-10, keygen biotech, China) was used to validate the interaction between mRNA and miRNA. pGL3 vectors were constructed containing either WT or mutant(Mut) 3’-UTR region with the putative miRNA bindingsite of mRNA of DUSP4. The vectors were then transfected into 293T cells with or without a miR-206-5p mimic. The activities of firefly luciferase values were measured 48 h after transfection by a microplate reader (SpectraMax iD5, MD, USA), and were normalized to Renilla luciferase values.

### Animal

All mice were purchased from Biotech-ΥueKang Life Science(shanghai)Co., Ltd maintained under specific pathogen-free conditions, and had free access to food and water. All animal experimental procedures were approved by the Animal Research Committee of Shanghai Jiao Tong University Affiliated Sixth People’s Hospital (SYXK2021-0028, Shanghai, China). A total of 25 adult female KM mice were randomly divided into 5 groups: [1] normal group, [2] tail-suspension + PBS injection group, [3] tail-suspension + iMSC-EV treatment group, [4] tail-suspension + miR@DT-Lipo treatment group, and [5] tail-suspension + miR@DT/iMNV treatment group. Tail suspension was performed for 8 consecutive weeks to establish an OSP model through prolonged weightlessness and disuse. Subsequently, the suspended mice were intravenously injected with 100 µL of PBS, iMSC-EV (1 × 10⁸ particles), miR@DT-Lipo, and miR@DT/iMNV (1 × 10⁸ particles) via the tail vein. The injections were administered every three days for a duration of 6 weeks. All mice were housed under specific pathogen-free (SPF) conditions. The tail skin condition was regularly checked, and a 12-hour light/12-hour dark cycle was maintained with free access to food and water. After 6 weeks of treatment, the mice were sacrificed, and bilateral femurs and muscles were collected for subsequent experimental evaluations. All surgeries were performed under anesthesia by isoflurane, and all efforts were made to minimize suffering.

### Biodistribution of miR@DT/iMNV in vivo

Each mouse was injected intravenously with DiR-labelled iMSC-EV/BT-Lipo/MT-Lipo/DT-Lipo/ miR@DT/iMNV at a dose of 1 mg/mL and 0.2 ml. After different time intervals, the mice were performed euthanasia. We isolated the major organs (heart, liver, spleen, lung, kidney, bones and muscles) to analyze the fluorescence intensity using Bruker Xtreme (Bruker Corp., Billerica, MA, USA, Germany).

### Cytotoxicity assays in vitro

Mouse BMSCs and MuSCs were used to access the cytotoxicity of miR@DT/iMNV with a CCK-8 kit (C0005, TargetMol, Boston, MA, USA). Different cells were incubated with PBS/iMSC-EV/miR@DT-Lipo/ miR@DT/iMNV for 24/48/72 h, then the cell viabilities were detected according to the protocols.

### In Vitro cellular uptake

A fluorescence microscope was used to investigate cellular uptake behavior. BMSCs and MuSCs cells were seeded overnight in 24-well plates. Subsequently, Lipo, iMSC-EV, BT-Lipo/MT-Lipo, miR@DT-Lipo or miR@DT/iMNV were incubated with cells for 2 h. Following this, the cells were washed and fixed, with the nucleus stained with DAPI before being subjected to fluorescence microscopy (Olympus, Japan).

### Enzyme-linked immunosorbent assay (ELISA)

To determine the cytokine concentrations in serum, mouse blood samples from the ophthalmic venous plexus were obtained after anesthesia. The serum concentrations of biochemical bone markers were detected with ELISA kits according to the protocols, including P1NP, TRAP5b, BAP (P1NP, MU30602; TRAP5b, MU30923; BAP, MU30270; Bio-Swamp, Wuhan, China), β-CTX (ED-22443, AMOY LUNCHANGSHUO BIOTECH, Co., Ltd., Xiamen, China) and OCN (OCN ELISA kit, Shanghai Hengyuan Biological Technology Co., Ltd., Shanghai, China).

### H&E staining

Femur bone samples, GAs and other organs (liver, heart, lung, kidney and spleen) collected from the different groups were fixed in ice-cold 4% paraformaldehyde for 24 h. Bone samples were decalcified for 3 weeks with 10% ethylene diamine tetraacetic acid (EDTA). Each organ was embedded in paraffin then sliced into 4-µm sections and subjected to hematoxylin and eosin staining. 

### Hindlimb grip strength assessments

Hindlimb grip strength was measured by using a customized grip strength meter for mice (Taixing experimental instrument factory, China) before mice sacrifice. Hindlimb grip strength assessments were performed three times by the same individual. The maximum force (N) was selected.

### Treadmill running test

The treadmill running test was conducted to assess muscular function and endurance capacity. Following a 5-minute acclimatization period at 5 m/min, the test protocol began at an initial speed of 10 m/min with 0° inclination. The speed was incrementally increased until volitional exhaustion. Exhaustion was defined as the inability of the animal to maintain pace despite gentle mechanical prodding for more than 10 s. The primary endpoints included both the total running time and the running distance. The test was performed in a climate-controlled environment, and all animals were familiarized with the treadmill apparatus on two separate occasions 48 h prior to the formal test to minimize stress.

### Pole-climbing test

The pole-climbing test was employed to evaluate muscular function and limb coordination. A vertical wooden pole (diameter: 2.5 cm; height: 60 cm) was wrapped with a non-slip mesh to facilitate grip. The time required for the mouse to complete the entire climbing process and the number of errors occurring during climbing were recorded. The test arena was softly padded to prevent injury upon potential falls.

### Immunofluorescence staining

Fixed RAW264.7, BMSCs, MuSCs/myotubes and the sections were permeabilized with 0.3% PBST for 15 minutes, followed by blocking with 5% BSA for 1 hour for immune staining for 15 min at RT, followed by incubation with primary antibody against iNOS (1:500, GB11119, Servicebio), Arginase1 (1:500, GB115724, Servicebio), IL-6 (1:200,ab233706, abcam), OCN (1:200, GB11233, Servicebio), OSX (1:500, GB111900, Servicebio), Pax7 (1:100, bs-22741R, Bioss), Myod1 (1:100, ab203383, Abcam, UK), eMyHC (1:300, F1.652, DSHB), fast MyHC (1:1000, GB112130, Servicebio), slow MyHC (1:1000, GB112131, Servicebio), laminin (1:50, ab11575, Abcam), at 4°C overnight and labeled with Alexa Fluor594-preabsorbed goat anti-rabbit IgG (ab150084, Abcam, 1:500), AlexaFluor 488 AffiniPure F(ab’)2 Fragment Goat Anti-Rabbit IgG (111-546-003,Jackson ImmunoResearch, 1:500) respectively for 2 h at room temperature. Next, the nucleus was stained with DAPI.

### Micro-CT analysis

Femurs were fixed using 4% paraformaldehyde for 1 day and scanned using a Skyscan 1172 (BrukerBioSpin, Belgium). The trabecular region starting from 0.15 mm below the growth plate and extending proximally to 0.4 mm was selected to collect the trabecular bone parameters. The structure image slices were then reconstructed into three-dimensional images.

### Statistical analysis

Numerical data are presented as the mean ± SD. One-way ANOVA for three or more groups and Student’s t-test for two groups were performed for statistical analysis using GraphPad Prism 10.1.2.

## Supplementary Information

Below is the link to the electronic supplementary material.

Supplementary Material 1

## Data Availability

No datasets were generated or analysed during the current study.
