# Supplementary material for: Dual-targeted hybrid nanovesicles coordinated bone-muscle regeneration via regulating the DUSP4/p38 MAPK pathway to reverse osteosarcopenia
Source: J Nanobiotechnology. 2026 Mar 16;24:385. doi: 10.1186/s12951-026-04279-4 (PMC13104562; doi:10.1186/s12951-026-04279-4)
Supplement: Supplementary file 1 — Supplementary Material 1 [file 12951_2026_4279_MOESM1_ESM.docx]

Supporting Information

**Dual-Targeted Hybrid Nanovesicles Coordinated Bone-Muscle Regeneration via Regulating the DUSP4/p38 MAPK Pathway to Reverse Osteosarcopenia**

*Benchi Che, Yongzhi Cui, Zhengsheng Chen, Yanchun Gao, Lei Luo, Kaiwen zheng, Jiashuo Liu, Yu Xiang, Jiaqi Cheng, Yuanyuan Guo*, Qing Li* and Dehao Fu**


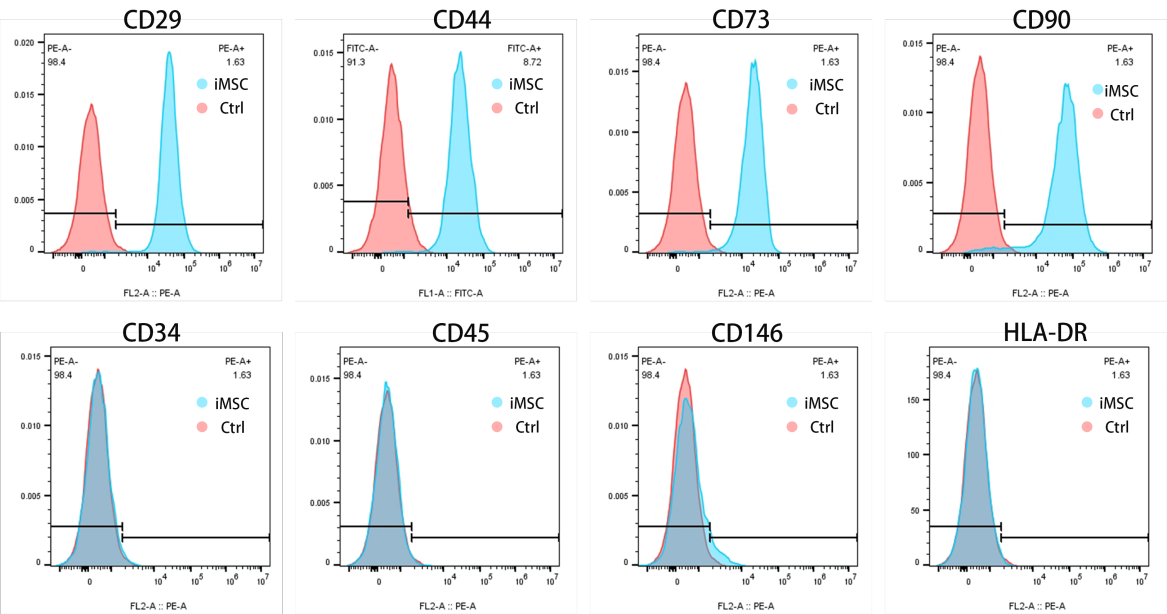


**Figure S1.** Expression of positive markers (CD29, CD44, CD73, CD90) and negative markers (CD34, CD45, CD146, HLA-DR) on the surface of iMSCs.


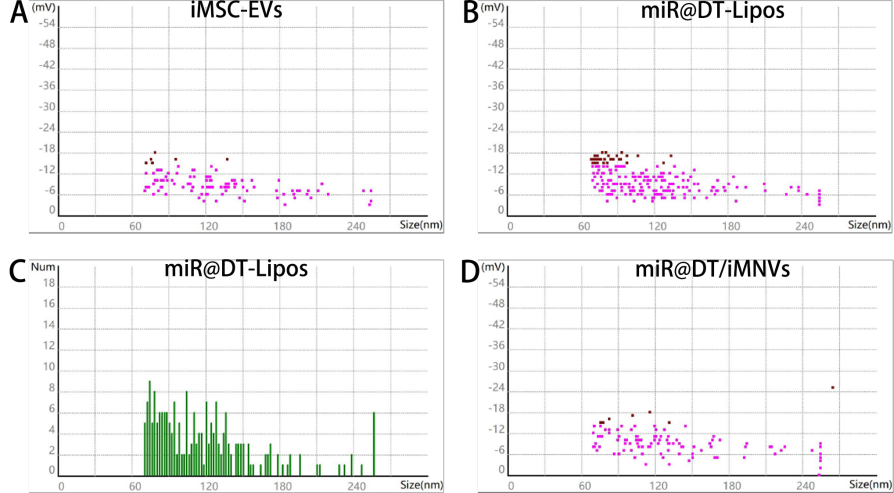


**Figure S2.** (A) Zeta potential of iMSC-EVs. (B) Zeta potential of miR@DT-Lipo. (C) Particle size analysis of miR@DT-Lipos. (D) Zeta potential of miR@DT/iMNVs.


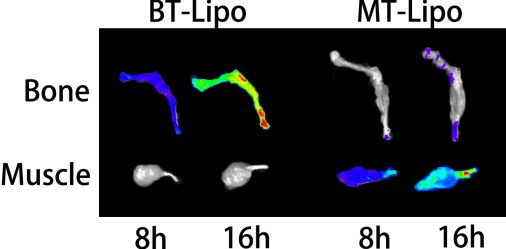


**Figure S3.** Biodistribution of DiR-labeled BT-Lipo and MT-Lipo.


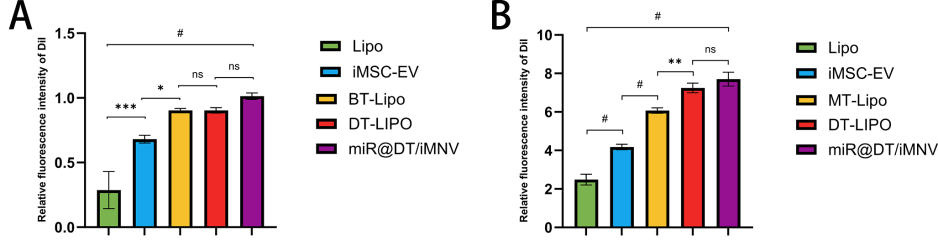


**Figure S4.** (A) Quantification of the immunofluorescence intensity of Dil-labeled different nanoparticles in BMSCs (n=3). (B) Quantification of the immunofluorescence intensity of Dil-labeled different nanoparticles in MuSCs (n=3). *p < 0.05, **p < 0.01, ***p < 0.001, and #p < 0.0001.


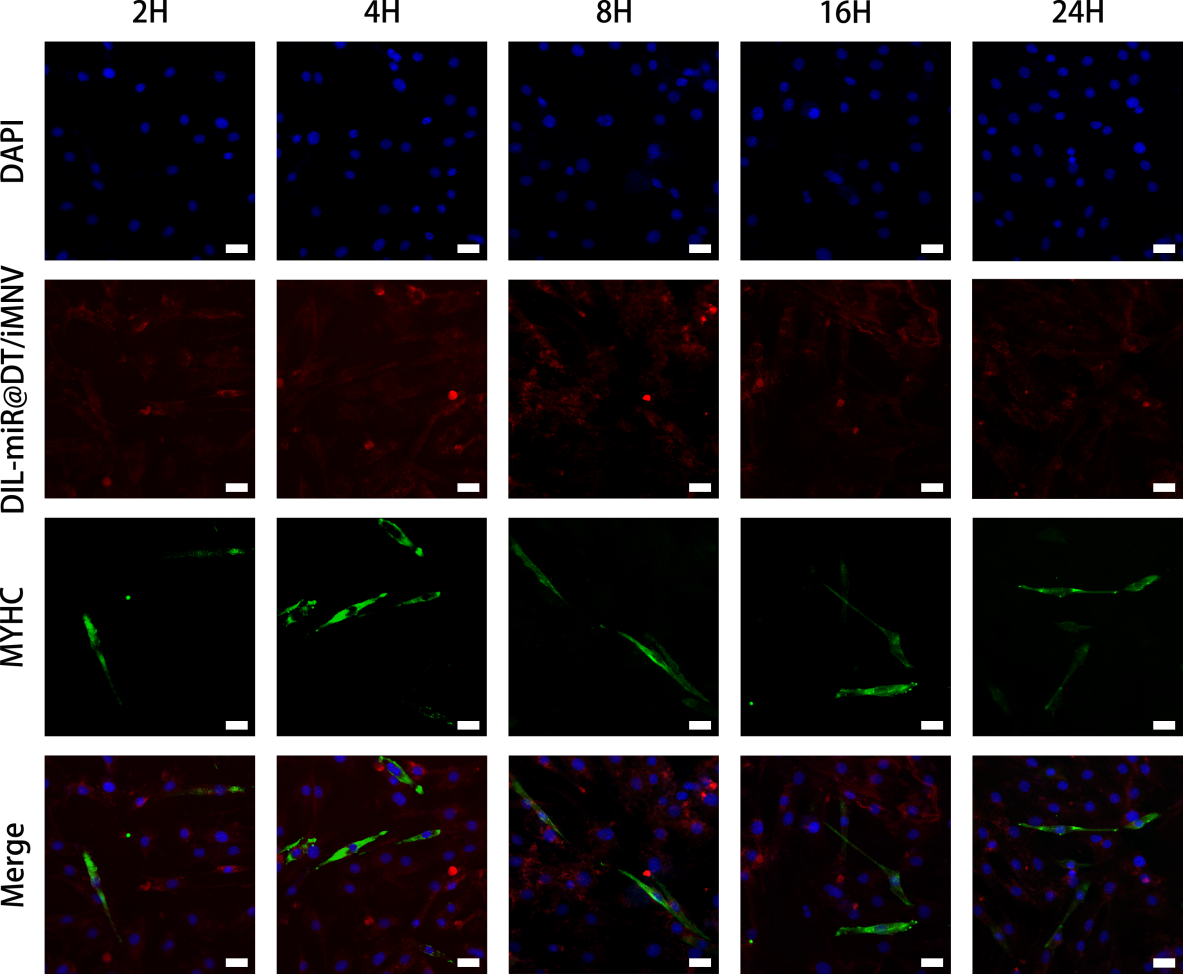


**Figure S5.** Immunofluorescence images of myotube uptake of miR@DT/iMNV at different time points. Scale bar: 20 μm.

**
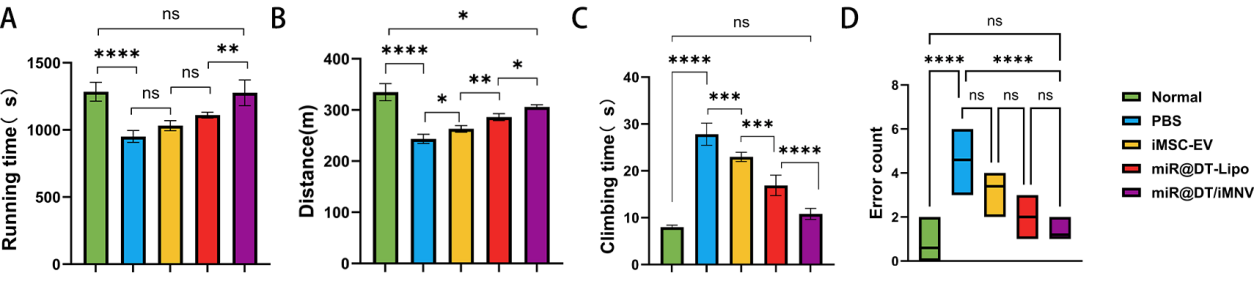
**

**Figure S6.** (A) Exercise duration in mouse treadmill experiments (n=5). (B) Running distance in mouse treadmill experiments (n=5). (C) Time consumption in mouse pole-climbing experiments. (D) Error Counts in Mouse Pole-Climbing Experiments (n=5).*p < 0.05, **p < 0.01, ***p < 0.001, and ****p < 0.0001.


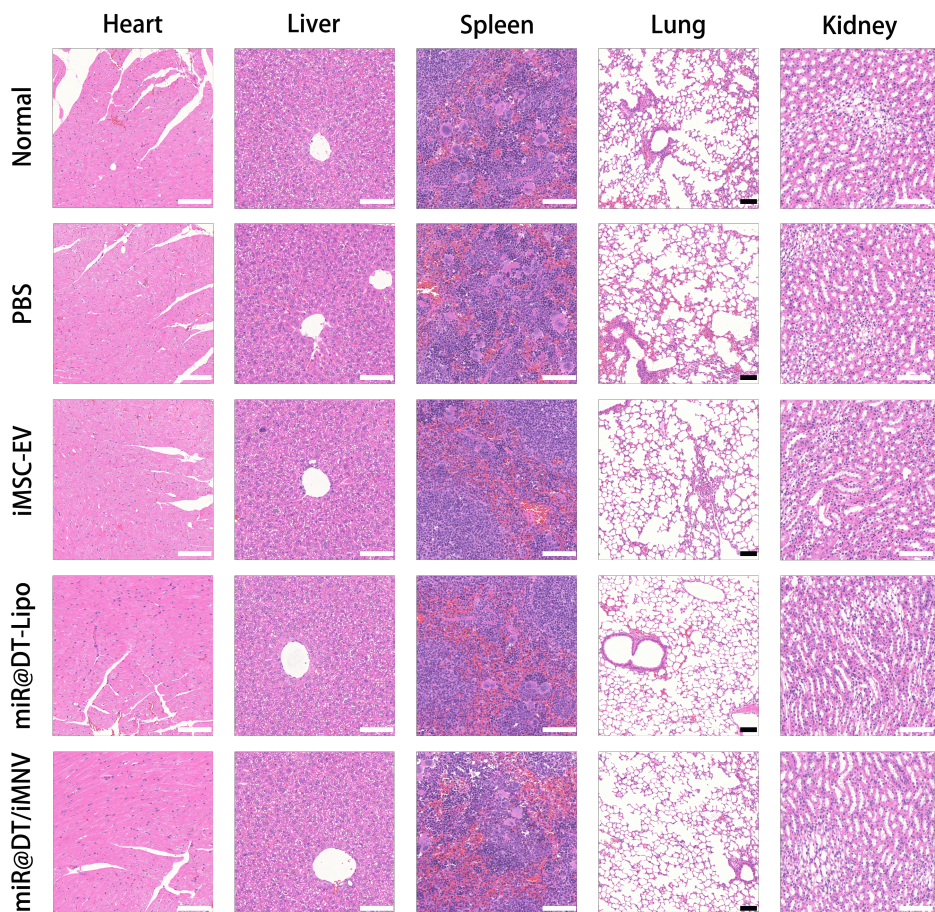


**Figure S7.** H&E staining images of major organs after different treatments. Scale bar: 100 μm.
